# Shifting reef restoration focus from coral survivorship to biodiversity using Reef Carpets

**DOI:** 10.1038/s42003-024-05831-4

**Published:** 2024-01-31

**Authors:** Yael B. Horoszowski-Fridman, Ido Izhaki, Sefano M. Katz, Ronen Barkan, Baruch Rinkevich

**Affiliations:** 1 Israel Oceanographic and Limnological Research, Tel-Shikmona, Haifa, 31080 Israel; 2 Department of Evolutionary and Environmental Biology, Faculty of Natural Sciences, University of Haifa, Haifa, 31905 Israel; 3 The School of Marine Sciences, Ruppin Academic Center, Michmoret, 40297 Israel; 4 Pacific Blue Foundation, PO Box 13306 Suva, Fiji Islands

**Keywords:** Restoration ecology, Tropical ecology

## Abstract

To enhance the practice of farmed-coral transplantation, we conducted a trial of an approach called “*Reef Carpets*” (RC), which draws inspiration from the commercial turf-grass sod in land-based lawn gardening. Three 8.4m^2^ RCs were established on a sandy seabed, containing preselected combinations of branching corals (*Acropora* cf. *variabilis*, *Pocillopora damicornis, Stylophora pistillata*) with nursery recruited dwellers, and were monitored for 17-months. Corals within RCs grew, supported coral recruitment and offered ecological habitats for coral-associated organisms. While the unstable sediment underneath the RCs increased corals’ partial mortalities, corals managed to grow and propagate. The extent of fish and gastropods corallivory varied among the coral species and planulation of *Stylophora* transplants was significantly higher than same-size natal-colonies. The RCs provided conducive environments for fish/invertebrate communities (183 taxa), and each coral species influenced specifically species-diversity and reef-associated communities. Even dead corals played crucial roles as habitats for reef biota, sustaining >80% of the RCs diversity; hence, they should not be considered automatically as indicators of failure. RCs scaled-up reef restoration and generated, in short periods, new reefs in denuded zones with enhanced biodiversity. Yet, RCs employment on soft-beds could be improved by using more structured artificial frameworks, requiring further research efforts.

## Abstract (web summary)

A new restoration approach reveals that planting ‘Reef Carpets’ instantly generates ecological habitats for coral-associated organisms and supports coral recruitment, providing an innovative tool to scaleup reef restoration and biodiversity benefits.

## Introduction

Coral reef ecosystems provide livelihood, food, and socio-cultural services to hundreds of millions of people around the world^1^, while sustaining one of the most diverse biological environments^2,3^. Yet, adverse climatic changes together with abiding anthropogenic impacts impede coral reefs’ resilience and natural adaptation^4–6^. Pollution, coastal development, overexploitation of reef resources, destructive fishing methodologies, seawater temperature rise, and ocean acidification are only part of the list of drivers that have made reefs one of the most threatened ecosystems on Earth^4,7^. Due to the limited effectiveness of traditional management approaches, such as Marine Protected Areas and fishing restrictions, in preventing widespread degradation of coral reefs (e.g., in refs. ^5,8–11^), active reef restoration has become a central focus in reef conservation efforts. One extensively investigated approach is known as “coral gardening”^12–15^.

Coral reefs, often referred to as the “rain forests of the sea”, share numerous ecological and functional properties with forest ecosystem^16,17^. As a result, the coral gardening approach integrates various principles from forest restoration, creating a toolbox for coral restoration that relies on ecological engineering approaches^18–21^. The coral gardening approach consists of two successive phases: (1) Cultivating a large number of coral colonies through sexual and asexual propagation in underwater nurseries until they reach suitable sizes, and (2) Outplanting the nursery-raised corals onto degraded reefs. The first phase has undergone extensive research^22–24^, demonstrating the ability to cultivate new coral colonies of >100 species using various nursery prototypes worldwide^25–29^. In contrast, the transplantation phase has received limited research attention, so many theoretical and practical aspects are yet to be studied.

Farmed-coral transplantation still faces several challenges. The current predominant method involves individually attaching grown colonies, which is labor-intensive and time-consuming, making it suitable only for small-scale efforts. Additionally, in many degraded reef areas, the substrates are no longer suitable for coral attachment due to factors such as dynamite fishing, sedimentation from land reclamation, or phase shift events^30–33^. Furthermore, the current practice of individual colony-planting overlooks one of the significant advantages of underwater coral nurseries compared to sterile terrestrial nurseries, following which the underwater nurseries have surfaced as platforms for recruiting reef-associated organisms^34–37^. Since reef-associated fauna is also under significant threat from degradation^2,38^, incorporating these recruited organisms alongside farmed colonies could enhance biodiversity in transplanted reefs. However, transplantation methods that consider the nursery-associated reef biota are not yet available.

To address above mentioned limitations and expand the active reef restoration toolbox, this study aims at the restoration of coral reefs on various unstable soft bottoms (where natural restoration processes usually fail; e.g., in refs. ^32,39,40^.) where coral reefs flourished in the past or where only patch reefs were found. Here we introduce a new transplantation method called “*Reef Carpets*” (RC) inspired by commercially available turf-grass sod units used for establishing or repairing lawns on land, aiming to provide an unorthodox approach to coral reef restoration. The RC concept builds upon the previously introduced *Coral Carpet* notion^41^. Yet, unlike the Coral Carpet which consists solely of coral transplants, the RC approach includes the integration of reef fauna that naturally develops among and around the growing corals, allowing the immediate formation of a structure resembling a natural reef (i.e., a “Reef Carpet”).

RCs of three branching species (*Acropora cf. variabilis, Pocillopora damicornis, Stylophora pistillata*) were cultivated and raised at the coral nursery in Eilat (Red Sea). These RCs, along with the associated reef biota acquired during the nursery phase, were then deployed on an unstable sandy substrate. Over a monitoring period of 17 months, their performance was assessed, including survival measures, partial tissue loss, reproductive outputs, and impacts from corallivores. Further, we studied some ecosystem engineering impacts of transplants on reef-associated species by observing the natural recruitment of reef fauna, as well as the structure and species diversity of the developing fish and invertebrate communities in the RCs.

## Results

### Overall outcomes: the development of reef oases

**Fig. 1:**
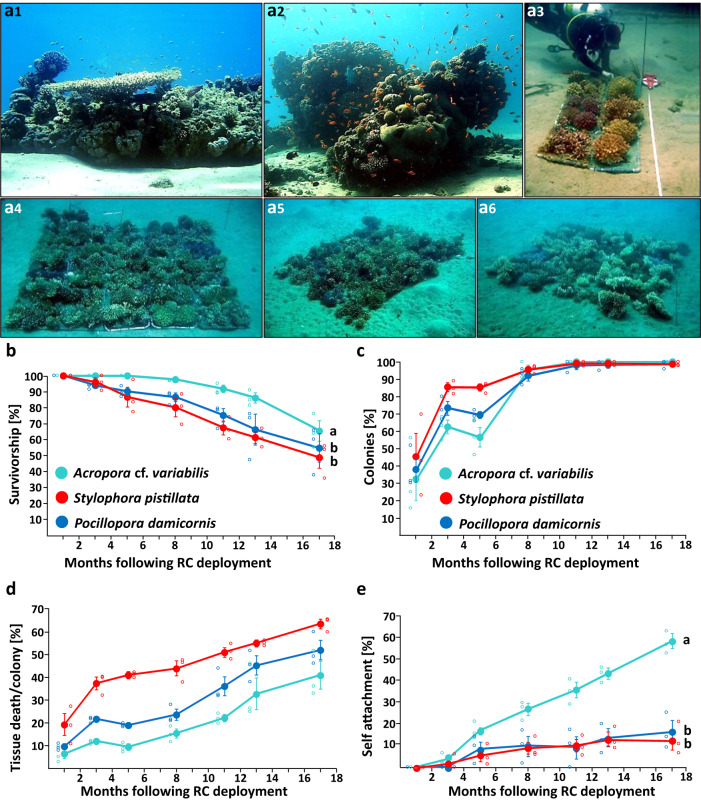
The construction of Reef Carpets (RCs) on sandy substrates and 17-months follow up of the 3 RCs established. Deployment of the Reef Carpets plots on a soft-bottom substrate, to restore the typical reef structures in the area (**a1**, **a2**). **a3–a6** An RC plot at: **a3** construction; **a4** immediately after deployment; a5) 7 months following deployment; a6) 17 months following deployment; **b** Survival (%) of transplants; **c** Percentages of colonies exhibiting partial mortality cases; **d** Partial mortality magnitude (% tissue loss/colony); **e** Self-attachment. Results are mean ± SE. A mixed-model ANOVA was performed for each panel. Letters on the right side denote statistically significant groups (Tukey post-hoc tests, *p* < 0.05). **a1** and **a2**—courtesy by S. Shafir.

Each of the three RCs, assembled and transplanted in the middle of a large sandy area (Fig. 1a4–a6), immediately developed into a vibrant reef environment. Despite the presence of movable sediment beneath the RCs, which caused partial colony mortalities followed by repair and subsequent damage, a significant number of corals managed to survive, flourish, and even participate in local coral reproduction. The ecosystem engineering effects of the RCs became evident rapidly as they stimulated the establishment of a diverse reef-associated community in all units (183 fish and invertebrate taxa, recorded by visual censuses; Tables S2 and S3). These RCs provided settlement contingency to numerous coral recruits that appeared among the corals (a total of 137, 111, and 73 new coral recruits spotted 1-, 3-, and 5-months post-transplantation, respectively; RC1-RC3 combined; Table 1), and settlement opportunities to coral-obligate organisms such as crustaceans (e.g., *Cymo*, *Trapezia* and *Tetralia* species) that specifically settled in distinct coral hosts. The RCs further provided ecosystem services when catering nursery sites for a variety of coral reef inhabitants, including juvenile fish (e.g., *Dascyllus trimaculatus*, *Chromis viridis*) and for attached egg masses (fish and cephalopods clutches, non-identifiable in situ). Despite the limited dimensions of the RC plots, they were sufficient for the establishment of a total of 11 *Labroides dimidiatus’* cleaning stations. The plots also became permanent sites of large predators such as *Pterois* that often preyed on *C. viridis* aggregates, which homed *Acropora* colonies, and were often visited by roving fish. Fish species such as *Coris aygula* and *Thalassoma lunare* were observed foraging for the corallivorous gastropods *Drupella cornus* and *Coralliophila sp*. The RCs also became the homes for grazers, attracting a variety of sea urchin species such as *Diadema setosum*, *Echinometra mathaei*, and *Tripneustes gratilla elatensis*, as well as fish (e.g., *Chrysiptera unimaculata*, *Pomacentrus trilineatus*) feeding on growing algae within RCs. Furthermore, the dead coral colonies were transformed into hubs for other reef organisms, providing habitats for a wide variety of typically cryptic and well-camouflaged species (e.g., Majoidea and Pilumnidae decapods). They also became substrates for the recruitment of sessile organisms such as tunicates (e.g., *Polycarpa mytiligera*, *Herdmania momus*, *Botrylloides sp*.), sponges (e.g., *Cliona sp*., *Mycale fistulifera*), and bivalves.

**Table 1:** Coral settlement on the 35 RC units (3 plots), 1-, 3-, and 5-months post RC assemblages, spotted on dead coral skeletons and on the RC trays and nets. Further details in Table S11.

Months after the RC installment		RC units with new settlements	Number of new Coral recruits	Recruits seen on:	
				RC coral skeletons	RC framework
1	RC 1	9	28	3	25
	RC 2	9	22	5	17
	RC 3	20	87	9	78
3	RC 1	16	55	10	45
	RC 2	19	31	15	16
	RC 3	11	25	5	20
5	RC 1	13	23	10	13
	RC 2	14	28	19	9
	RC 3	11	22	15	7

### Coral attributes

The survival of the 132 *A*. cf. *variabilis*, 212 *P. damicornis* and 364 *S. pistillata* transplanted colonies, following 17 months in the three RCs, averaged (mean ± SE) 65.3 ± 6.2%, 54.6 ± 8.6% and 48.7 ± 6.5%, respectively (Fig. 1b), exhibiting significantly different survival rates (mixed-model ANOVA, *F*_12,36_ = 4.076, *p* = 0.001; Fig. 1b) between the mostly surviving *A*. cf. *variabilis* and the other two species (Tukey post-hoc tests, *p* > 0.05). The first mortality case in *A*. cf. *variabilis* was recorded only eight months post-transplantation. The median post-transplantation lifespans of *A*. cf. *variabilis*, *P. damicornis*, and *S. pistillata* were 17, 13, and 11 months, respectively, and the probability of survival was significantly different for each of the three species (Kaplan–Meier analysis, Mantel–Cox *log-rank* test, *χ*^*2*^ = 7.087, df = 2, *p* = 0.029; Table S4; Fig. S2).

The number of colonies disappearing from the RCs after 17 months was marginal, ranging from 4.2 ± 1.3% (*S. pistillata*) to 6.2 ± 3.4% (*P. damicornis*), and did not differ significantly between the three species (mixed-model ANOVA, *F*_12,36_ = 0.746, *p* = 0.698; Fig. S3).

Partial colony mortality was a common occurrence in all three coral species and gradually increased over time. Upon transplantation, several transplants already exhibited varying degrees of partial mortality (32.3 ± 12.2% of *A*. cf. *variabilis* colonies, 37.8 ± 7.0% of *P. damicornis* and 45.3 ± 13.2% of *S. pistillata* showed 6.4 ± 2.0%, 9.6 ± 1.0% and 19.3 ± 4.7% partial mortality/colony, respectively). Considering that partial mortality can potentially affect coral performance^42,43^, especially when transplanted into the challenging environment of Kisuski Beach, we first explored whether the extent of partial mortality at transplantation influenced subsequent colony survival, revealing no significant difference in survival between partially impacted vs. intact colonies at transplantation onset (mixed-model ANOVAs—*A*. cf. *variabilis*: *F*_2.035,8.139_ = 3.755, *p* = 0.069; *P. damicornis*: *F*_4.173,16.690_ = 0.882, *p* = 0.499; *S. pistillata*: *F*_4.582,18.330_ = 1.753, *p* = 0.176; Fig. S4). After the deployment of the RCs, the sandy substrate beneath the trays continuously shifted, passing through the nets (Fig. 1a4–a6), contributed to the occurrence of partial mortality and resulted in a significant increase in the percentage of colonies experiencing partial mortality over time (mixed-model ANOVA, *F*_2.739,16.432_ = 79.304, *p* < 0.001), equally affecting the three species (*F*_5.477,16.432_ = 1.863, *p* = 0.152; Fig. 1c). After a period of 17 months, partial mortality was observed in 96%–100% of the colonies. Its extent, which increased significantly over time (mixed-model ANOVA, *F*_3.505,21.028_ = 80.021, *p* < 0.001) averaged at the end of observations 41.0 ± 6.0%, 52.1 ± 4.1% and 63.7 ± 1.7% in *A*. cf. *variabilis*, *P. damicornis*, and *S. pistillata*, respectively (*F*_7.009,21.028_ = 1.728, *p* = 0.156; Fig. 1d).

Despite the adverse impacts of the moving sediment, the colonies on the RC structures persisted in growing and spreading. Self-attachment of transplants to the RC frames increased significantly over time (mixed-model ANOVA, *F*_6,36_ = 95.994, *p* < 0.001; Fig. 1e), at a higher rate in *A*. cf. *variabilis* (*F*_12,36_ = 7.424, *p* < 0.001; Tukey post-hoc tests, *p* < 0.05), whereas the two pocilloporid species revealed comparable attachment rates (Tukey post-hoc tests, *p* > 0.05). After 17 months, 58.0 ± 3.3% of *A*. cf. *variabilis*, 16.2 ± 5.1% of *P. damicornis* and 12.5 ± 4.5% of *S. pistillata* were permanently attached to the RC sub-frames.

**Fig. 2:**
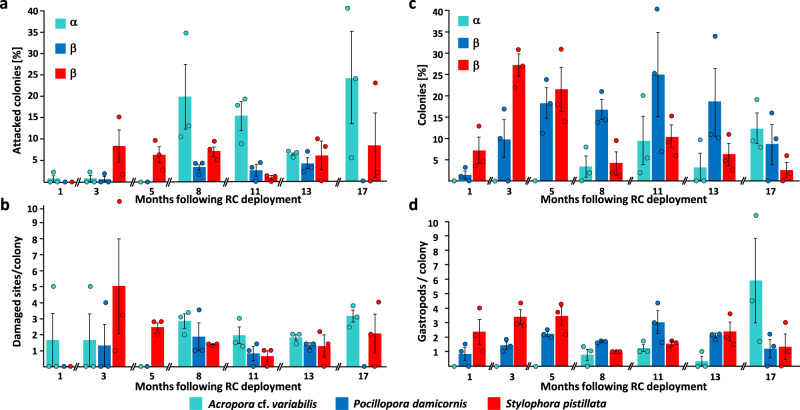
Fish and gastropods corallivory on coral colonies residing within Reef Carpets and numbers of residing predatory gastropods within coral colonies. Predation by fish and gastropods **a** Fish corallivory (%); **b** Average numbers of damaged sites per affected colony; **c** Transplants (%) with predatory snails; **d** Average numbers of predatory gastropod in affected colonies. Mean ± SE. In panels “**a**” and “**c**”, a mixed-model ANOVA was performed and significant differences between the coral species metrics are denoted by letters (α, β) on the left side in these panels (Tukey post-hoc tests, *p* < 0.05).

Corallivory by fish and gastropod snails was dynamic throughout the 17 months period and the extent of fish attacks on the corals’ tissue varied over time (mixed-model ANOVA, *F*_3.904,23.424_ = 6.932, *p* = 0.001; Fig. 2a). *A*. cf. *variabilis* and *S. pistillata* were found to be more susceptible to fish attacks compared to *P. damicornis* (*F*_7.808,23.424_ = 3.945, *p* = 0.005; Tukey post-hoc tests, *p* < 0.05), yet the number of colonies that showed fish bites did not exceed 24.1 ± 12.7% (recorded in *A*. cf. *variabilis* at last observation time-point). The number of fish bites per colony was comparable among the three species throughout the survey, fluctuating between 0.7 ± 0.3 to 5.1 ± 3.0 bites/attacked colony (mixed-model ANOVA, *F*_5.982,17.946_ = 1.523, *p* = 0.227; Fig. 2b). Foraging by corallivorous gastropods *Drupella cornus* and *Coralliophila sp*. further inflicted damages on the three species transplants (Fig. 2c), pocilloporid colonies were significantly more impacted (mixed-model ANOVA, *F*_12,36_ = 5.000, *p* < 0.001; Tukey post-hoc tests, *p* < 0.05), with no difference between the two species (Tukey post-hoc tests, *p* > 0.05). The combined numbers of these gastropods in foraged colonies varied between 0.3 ± 0.3 to 5.9 ± 2.9 gastropods/colony in *A*. cf. *variabilis*, 0.8 ± 0.4 to 3.0 ± 0.8 in *P. damicornis*, and 1.0 ± 0 to 3.4 ± 0.6 in *S. pistillata* (Fig. 2d). Mixed-model ANOVA analysis indicated a significant interaction between time and species (*F*_4.459,13.375_ = 3.991, *p* = 0.022), yet Tukey post-hoc tests showed that no pair of species was significantly different from each other.

### Larval release in *S. pistillata*

Planulae were obtained from 50% of natal and 68.3% (mean of RC1-RC3) of transplanted *S. pistillata* colonies (Table 2 and Fig. S5). Although the proportion of gravid colonies did not significantly differ between the two groups (One-sample *t*-test, *t*_2_ = 4.16, *p* = 0.053), significantly more planulae/colony were released by the RC transplants than by the natal colonies (RC1-RC3, 4.6 ± 1.7 planulae/transplant vs. 1.6 ± 0.6 planulae/natal colony; mean ± SE; One-sample *t*-test, *t*_2_ = 4.45, *p* = 0.047; Fig. S5).

**Table 2:** In situ planulae collections from naturally growing *Stylophora pistillata* colonies and from nursery-bred transplants in the Reef Carpet plots (RC1-RC3).

Colonies examined (*n*)		Planulae-releasing colonies (%)	Planulae collected/colony	Total planulae collected	Average planulae/colony ± SE	
			Min	Max		
Natal	16	50	0	9	26	1.62 ± 0.62
RC 1	20	70	0	16	82	4.10 ± 1.19
RC 2	20	60	0	24	75	3.75 ± 1.50
RC 3	20	75	0	38	118	5.90 ± 2.19

### The reef-associated communities

The existence of RC plots within the sandy area of Kisoski Beach attracted coral reef-associated species, leading to the emergence of new communities of coral inhabiting fish and invertebrates that were previously absent. We recorded in the RCs 66 fish taxa (Table S2) and 123 invertebrate taxa, out of which 39 were crustaceans (Table S3). Most fish taxa (71.2%; *N* = 44) were identified to the species level, 9.1% (*N* = 6) to genus, and 18.2% (*N* = 12) were assigned to the family level, altogether representing 47 species from 39 genera belonging to 19 fish families. The identification of the invertebrate specimens under field conditions required extensive effort. Only 32.5% (*N* = 40) invertebrate taxa were identified to the species level, 12.2% (*N* = 15) to the genus level, 16.3% (*N* = 20) to families, and the remaining invertebrate taxa were classified to lower taxonomic ranks [Infraorder: 14.6% (*N* = 18); Order: 4.9% (*N* = 6); Subclass: 0.8% (*N* = 1); Class: 6.5% (*N* = 8) ; Phylum: 12.2% (*N* = 15)], representing 40 species, 50 genera and 51 families. For the 39 crustacean taxa, 35.9% (*N* = 14) were identified to the species level, 5.1% (*N* = 2) to genus, 7.7% (*N* = 3) to family, 46.2% (*N* = 18) to Infraorder, and 5.1% (*N* = 2) to the order level, representing 14 species from 13 genera belonging to 14 families of crustaceans.

**Fig. 3:**
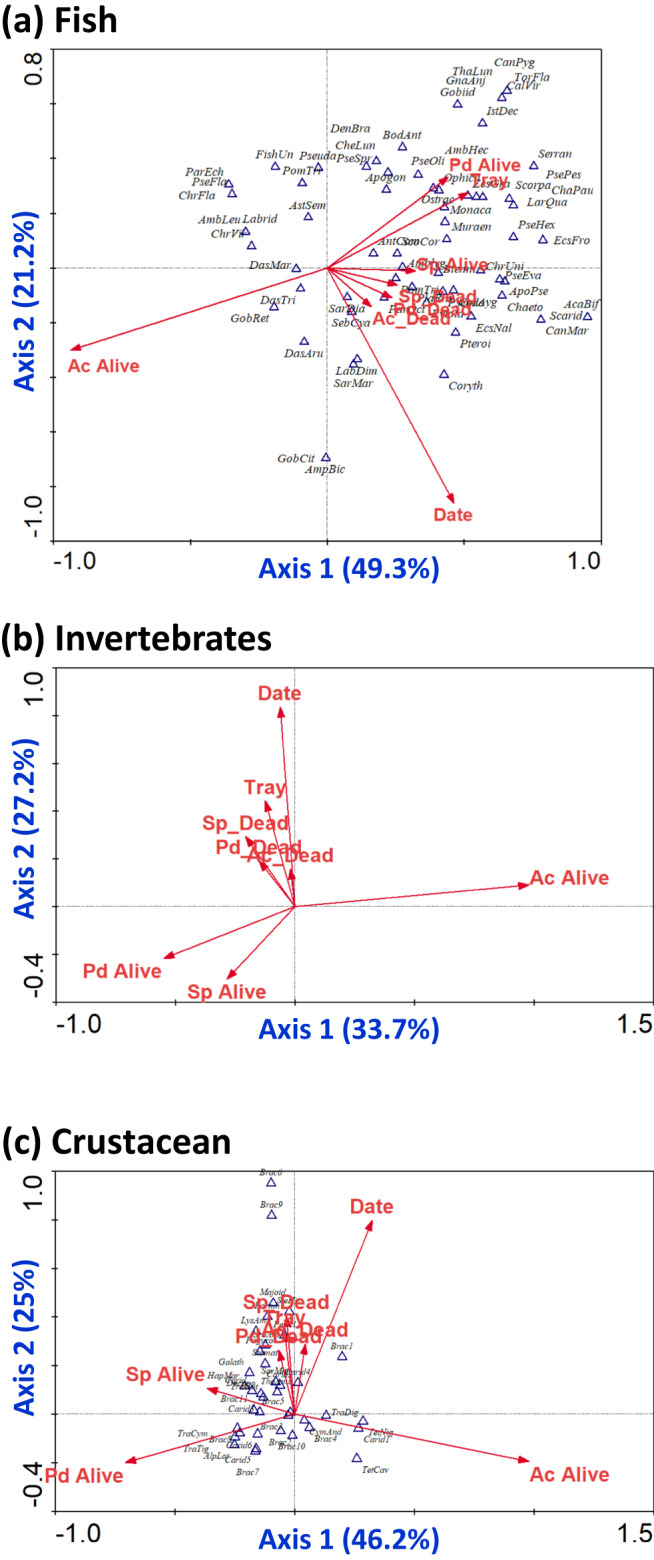
Species-environment b-plots based on a canonical correspondence analysis (CCA) for the effect of environmental factors (arrows) on RC community structure (species/taxa represented by triangles). **a** Fish species/taxa. Eigenvalues: axis 1 = 0.530; axis 2 = 0.228. The variance explained by each axis (%) is shown. **b** Invertebrate species. Eigenvalues: axis 1 = 0.466; axis 2 = 0.375. The variance explained by each axis (%) is shown. For clarity, only environmental variables (arrows) are shown (see Fig. S7 for the complete diagram with the 123 taxa). **c** Crustacean species. Eigenvalues: axis 1 = 0.651; axis 2 = 0.353. In each ordination diagram, the variance explained by each axis (%) is shown. Environmental variables: time in months since RC transplantation (*Date*); spotted in a live *A*. cf. *variabilis*, *P. damicornis*, or *S. pistillata* colony (*Ac Alive*, *Pd Alive*, and *Sp Alive*, respectively), in a dead colony of the before mentioned species (*Ac Dead*, *Pd Dead*, and *Sp Dead*, respectively), or at the understory (*Tray*). For a list of species abbreviations—see Tables S2 and S3.

The presence of the three coral species and the development of ecological niches had distinct impacts on the structure of the fish and invertebrate communities that formed at the RCs over time (Fig. 3).

As neither the RC nor the location within the RC (center/periphery) contributed to the structuring of the RC communities (Fig. S6), the three position variables (plot, center, periphery) were excluded from subsequent CCA analyses. Considering the fish community structure (Fig. 3a), axis 1 of the ordination was mainly affected by the coral species and the understory niche type (represented by the variable “tray”), with a strong effect of live *A*. cf. *variabilis* colonies, live *P. damicornis*, and the understory. Dead colonies of the three coral species had a comparable weight on the fish community structure. Axis 2 of the ordination was strongly influenced by the time scale since RC deployment. The distribution of fish taxa along the ordinates was not random; the two axes accounted for 70.5% of the variance in the species-location relation (Monte Carlo test, *F* = 7.250, *p* = 0.002). Three groups of fish assemblages emerged: 1) those associated with live *A*. cf. *variabilis* colonies, mostly coral-specialist species found among live corals (e.g., *Gobiodon reticulatus*, *Dascyllus aruanus*, *Dascyllus trimaculatus*); 2) species related to live *P. damicornis* colonies and the understory substrate, including species with strong habitat association with live corals (e.g., *Pseudochromis olivaceus*) and bottom-dwelling species (e.g., *Muraenidae*); 3) fish species associated with live *S. pistillata* colonies and dead coral skeletons (e.g., *Amblyglyphidodon*, *Ecsenius*, and *Pterois sp*.).

For the invertebrate communities (including crustaceans; Fig. 3b), live colonies of the three coral species had a substantial impact on axis 1 of the ordination, a strong effect of live *A*. cf. *variabilis*, followed by *P. damicornis* and *S. pistillata*. Like in the fish communities, axis 2 was strongly impacted by the time scale and was also affected by the dead coral colonies and the understory environment. Taxa distribution along the ordinates was not random, and the two axes accounted for 60.9% of the variance in the species-environment relation (Monte Carlo test, *F* = 25.563, *p* = 0.002). Three groups of species emerged: invertebrate species associated with live *A*. cf. *variabilis* colonies (e.g., *Tetralia cavimana*), species associated with live pocilloporid colonies (e.g., *Trapezia cymodoce*), and species associated with dead coral skeletons or the RC framework underneath the colonies (e.g., *Mycale fistulifera*, and most ascidians and sponge morphospecies recorded).

Focusing on the crustacean community (Fig. 3c), axis 1 was mainly affected by live *A*. cf. *variabilis* colonies, followed by live *P. damicornis* and *S. pistillata* colonies. As with the fish and all invertebrates, axis 2 was strongly impacted by time with a similar effect of dead coral colonies and the understory substrate. Taxa distribution was not random, and the two axes accounted for 71.2% of the variance in the species-environment relation (Monte Carlo test, *F* = 35.251, *p* = 0.002). Four groups of crustacean species were defined, reflecting the preferences of many coral-dependent crustaceans for a specific coral host:^37,44^ species attracted to live *A*. cf. *variabilis* colonies (e.g., *Tetraloides nigrifrons*, *Tetralia cavimana*), to live *P. damicornis* colonies (e.g., *Alpheus lottini*), to live *S. pistillata* colonies (e.g., *Hapalocarcinus marsupialis*), and species associated with dead coral skeletons or the RC underlying substrate (e.g., *Lysmata amboinensis*).

**Fig. 4:**
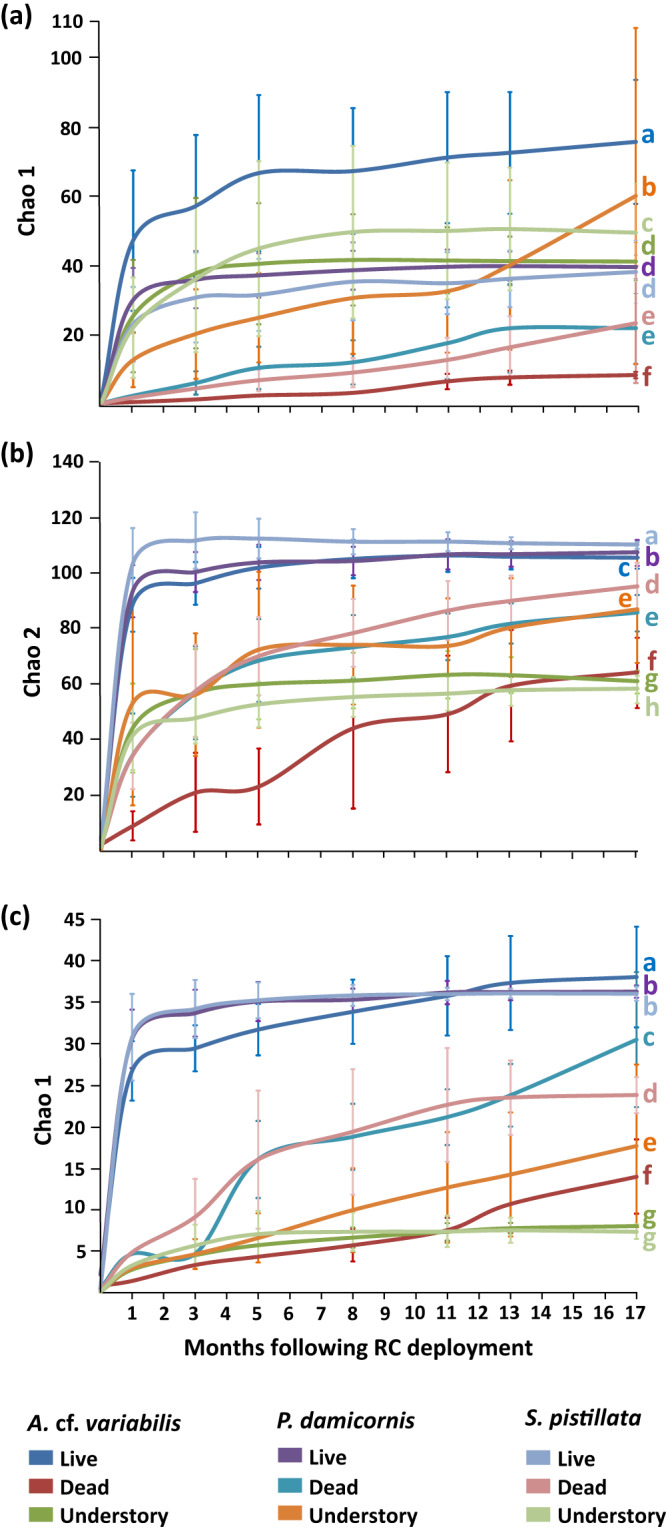
Species diversity based on Chao 1 and Chao 2 richness estimators ( ± SD) of reef-associated communities developing on the RC plots over 17 months. **a** fish diversity, **b** invertebrate diversity, **c** crustacean diversity associated with live colonies, dead colonies, and the understory spaces of *A*. cf. *variabilis*, *P. damicornis* and *S. pistillata* transplants. In each panel, diversity was compared with one-way ANOVAs. Letters on the right side denote statistically significant groups (Tukey HSD post-hoc tests *p* < 0.05; Tables S6, S8, S10).

After 17 months, the community diversity differed significantly between the three coral species (Fig. S8; Tables S5–S10) and between the specific habitats generated by the RCs (i.e., live colonies, dead colonies, and the understory compartment, for each of the coral species; one-way ANOVAs, Fish: *F*_8,5762_ = 853.435, *p* < 0.001; invertebrates: *F*_8,5762_ = 4302.382, *p* < 0.001; crustaceans: *F*_8,5762_ = 4571.741, *p* < 0.001; Fig. 4a–c; Tables S6, S8, S10).

In the fish community, the highest species diversity was associated with live *A*. cf. *variabilis* colonies (76 ± 18 species), followed by the understory environments of the three coral species (60 ± 48, 49 ± 14, and 41 ± 5 fish species for *P. damicornis*, *S. pistillata* and *A*. cf. *variabilis* understories, respectively; Fig. 4a). The *Acropora* understory microenvironment had comparable fish diversity to live *P. damicornis* and *S. pistillata* (39 ± 3 and 38 ± 9 fish species, respectively). The dead corals from the two pocilloporid species did not significantly differ (23 ± 17 and 22 ± 10 fish species for *S. pistillata* and *P. damicornis*, respectively), and the lowest fish diversity was attributed to dead *A*. cf. *variabilis* (8 ± 1 fish species).

Examining the invertebrate communities, the highest diversity was observed in live colonies of the three coral species, in terms of the overall community and among crustacean species (maximum of 110 ± 1, 107 ± 5, and 105 ± 4 total invertebrate species for live *S. pistillata*, *P. damicornis* and *A*. cf. *variabilis* colonies, respectively; maximum of 38 ± 6 crustacean species for live *A*. cf. *variabilis* colonies and 36 ± 1 crustacean for live *S. pistillata* and *P. damicornis* colonies; Fig. 4b, c). Lower diversity was recorded for dead pocilloporid corals and for *Pocillopora* understory (95 ± 7, 87 ± 19 and 86 ± 7 invertebrate species in dead *S. pistillata*, *P. damicornis* understory and dead *P. damicornis*, respectively; 30 ± 8, 24 ± 2 and 18 ± 10 crustacean species in dead *P. damicornis*, dead *S. pistillata*, and *P. damicornis* understory, respectively). The lowest species diversity was associated with dead *A*. cf. *variabilis* colonies (64 ± 13 invertebrate and 14 ± 4 crustacean species) and the *Acropora* and *Stylophora* understories (61 ± 4 and 58 ± 5 invertebrate species, respectively; 8 ± 0.1 and 7 ± 1 crustacean, respectively).

Dead Pocilloporid colonies harbored a substantial fraction of the invertebrate diversity, reaching 80-86% (invertebrate) and 66-83% (crustacean) of species diversity in live Pocilloporid corals. While obligate symbionts that depend on coral hosts for food and shelter, such as *Trapezia guttata* and *Hapalocarcinus marsupialis* were observed only on live corals, the dead colonies supported generalist species such as the omnivorous cleaner shrimps *Saron marmoratus*, and *Stenopus hispidus*. Moreover, three invertebrate taxa (belonging to Phyllodocida, Porifera, and Mollusca) were only present in dead colonies. Dead coral heads, thus, emerged as significant habitats for reef-associated fauna, with estimated species diversity ranging from 11% to 86% of the assessed diversity found in live coral. (Fig. 4a–c).

## Discussion

The continued degradation of coral reefs worldwide demand new restoration methods to enhance and broaden existing biodiversity^4–6^, one of which is the gardening approach^14,15,45^. This approach has been successful in propagating diverse coral species for restoration in various locations worldwide^15,29,46^. However, significant challenges remain in establishing large-scale restoration efforts, developing fast and effective transplantation methods^47–50^, and addressing coral transplantation on soft, movable substrates^32,39,40^. Additionally, current reef restoration practices often focus solely on corals^20,51^, overlooking the conservation needs of reef-associated fauna, which are also under threat and essential for biodiversity preservation. To address the issue of neglecting reef-associated fauna in restoration efforts, this study, that follows Golomb et al.^41^. study and the notion of ecological engineering approach^52^, has explored a new reef restoration tool named “*Reef Carpet*” (RC), that draws inspiration from the use of turf-grass sod units in lawn gardening. In the RC methodology, RC units are cultivated in coral nurseries and then assembled and transplanted as continuous units onto degraded areas to establish new reefs. Though direct quantitative comparisons cannot be made at this state between the outcomes of this study and those reported in the literature, especially concerning the mentioned variables, a significant advancement lies in the innovative aspect of this approach. Specifically, it aims to incorporate reef-associated fauna in restoration acts, with the purpose of enhancing restoration processes. The RC approach further offers a seemingly more efficient alternative to the labor-intensive practice of individually transplanting coral colonies, commonly employed in current restoration efforts^53,54^, promoting coral recruitment and facilitating increased sexual reproduction, while also creating additional ecological niches for invertebrates and fish. Consequently, it enhances reef fauna recruitment in both the coral nursery and restored areas, thereby bolstering the overall services provided by the reef ecosystem.

The choice of this site was influenced by the authority permit, that mandated its conduction at a challenging site, characterized by a hostile and continuously shifting soft sediment area, which makes it unfavorable for the proliferation of individual corals. This most probably led to a notable rise in partial mortality rates and a less than 65% survivorship of coral transplants after 17 months. Yet, this survival rate is reminiscent of the results observed in a different study that focused on coral outplanting on a degraded rocky reef substrate, located 1.5 km south of the present site in Eilat^55^. Comparable to this former study, the corallivory activity by fish and Muricid gastropods is almost half of the rate of fish corallivory recorded in Horoszowski-Fridman et al. ^55^. and minimal when compared with the last-decade voracious predation of *Drupella* and *Coralliophila* in the northern Red Sea^56–58^ and elsewhere, causing extensive coral mortality^59–62^. The presence of fish such as *Coris aygula* and *Thalassoma lunare* further contributed to the regulation of corallivore gastropod predation at the RCs. Interestingly, the decline in *A*. cf. *variabilis* survivorship and the increase in its partial mortality rates, first observed after eight months, coincided with the appearance of the corallivorous snails on *Acropora* colonies. Despite the challenging environmental conditions, the study observed high reproductive outputs in transplanted *S. pistillata* colonies compared to their natal colonies. This finding is reminiscent of previous studies that have documented extended periods (over 8 years) of enhanced larval production in nursery-bred transplants^63,64^, highlighting a long-term and continual effect of the nursery-rearing phase.

One of the notable outcomes of this study is the successful recruitment of reef-associated biota ( > 183 fish and invertebrate taxa) to the three RCs. Traditionally, the recruitment aspect has often been overlooked in reef restoration evaluations (yet, see refs. ^55,65,66^.). By using three coral-engineering species, each characterized by its unique species-specific 3D constructions^67^, a diverse array of new habitats for reef-dwelling organisms was created. These habitats included live colonies, dead colonies, and the biota inhabiting the understory, each specific to the coral species, resulting in an overall increase in biodiversity within the restored areas. Thus, the results underscore allogenic ecological engineering roles for the RCs, also characterized by dynamic and complex networks of biological interactions (e.g., “cleaning stations” by *Labroides dimidiatus*), emerged due to the creation of diverse open space compartments facilitated by the unique 3D properties of the branching coral species^37,67–69^. The ecological niches formed by both live and dead coral colonies, along with the diverse 3D assemblies created by the different coral species, have proven to be crucial in shaping community composition and promoting species diversity. This observation highlights the significance of structural relief in coral reef ecosystems, as many reef organisms heavily depend on these physical structures for shelter, foraging, and other ecological interactions^67,70–72^.

This study marks a notable shift in perspective by focusing on the reef services outcomes associated with dead coral colonies. Unlike previous studies that often regarded dead colonies as losses or failures in restoration efforts^29,73^, this research recognizes the ecological significance and potential contributions of these structures. Live and dead corals offer different resources for reef fauna^37,69,74,75^. For example, live corals provide nutritional sources such as live coral tissue, mucus, and the release of particulate organic matter, whereas micro/filamentous algae and detritus are abundant on dead coral surfaces, which also provide a more generous assortment of food resources. The live and dead coral colonies offer different shelter types and camouflage opportunities^69,76^, providing different settlement cues for reef-associated taxa^37,68^, and even different substrates for specialized burrowers and gall-forming species^38^, as for encrusting and sessile invertebrates. The surface covered by coral tissue is homogenous and somewhat simplified compared with the spatial heterogeneity of microhabitats provided by dead corals^44,77^. A degraded reef represents a chronic state in which biodiversity, 3D spaces, ecological networks, reef resilience, and reef services gradually decline over time. In contrast, our results demonstrate that the presence of dead coral colonies in transplantation efforts may introduce new and distinct dimensions to the rehabilitation processes by providing different niches compared to those offered by live colonies. Thus, together with live corals, dead colonies may increase the niche spectrum in the reef, not only for coral-specialists^44,71,75^, but also for generalists and non-coral-obligate taxa^77^. It is, however, of significant importance to assess the mortality levels of transplants to ensure that high levels of dead transplants do not hinder the overall trajectory of reef rehabilitation acts.

Furthermore, new 3D spaces are constructed within dead coral colonies and between-colonies areas that may host a wide range of reef-dwelling organisms^78,79^ that may sustain, as revealed in this study, up to 86% of the associated species diversity. Plaisance et al. ^3^. showed dead coral heads of four coral families (Pocilloporidae, Meandrinidae, Poritidae, Agariciidae) support substantial reef biodiversity. Wee et al.^37^. conducted a study on epifaunal invertebrate communities in nursery-grown corals, comparing live and dead fragments. The study revealed that dead fragments of *Pocillopora acuta* and *Platygyra sinensis* supported higher abundance and taxonomic richness of invertebrates and that Platygyra’s dead fragments exhibited higher diversity compared to live coral fragments. A study on decapod communities in live and dead *Pocillopora meandrina* colonies^77^ revealed substantial diverging species diversities, a result further supported by censuses on motile cryptofauna richness in other studies^75,78^. Similar conclusions were assigned to reef fish, revealing that moderate levels of coral mortality increase habitat heterogeneity and associated fish diversity^69,76,80^. The same implies in forestry, where dead habitat elements (e.g., dead trees, logs, coarse woody debris) are essential components in restoration schemes to increase structural diversity and biodiversity enhancement^78,81,82^. Thus, the additional 3D spaces constructed within dead corals and between-colonies areas should further be granted attention in reef restoration as the diversity and abundance of reef fauna are strongly linked to habitat complexity^71,72,74,83^.

In the present study, the RC approach has demonstrated its potential as a primary tool for reef restoration. Yet, there is an opportunity to complement it with a more structured artificial framework approach (such as the provision of suitable substrates for coral colony recruitments, boosting up natural recovery processes). In addition, it is advisable to use natural materials for the construction of RC. Thus, additional research is required to enhance the efficacy of Reef Carpets as a tool for coral reef restoration. This research should address various issues associated with the species utilized, the scalability of the approach, and the choice of substrate, interspecific and intraspecific interactions developing within the RC units, and the combined effects of species with different characteristics (colony morphology, life-history traits, growth rates, bleaching susceptibilities, etc.) on biodiversity enhancement. Indeed, the utilization of RC as a restoration tool offers promising benefits in terms of scale and targeted areas. It enables rapid coverage of bare, coral-depleted areas with coral assemblages, along with associated biota. Furthermore, the increased reproductive capacity of nursery-bred transplants can play a crucial role in enhancing coral reproduction within restored reefs and amplifying the overall outcomes of restoration efforts. By introducing genetically diverse and resilient individuals, the restored populations have a greater potential for adapting to future environmental changes, also helping to enhance the genetic diversity of previously degraded coral populations, and strengthening their ability to cope with various challenges in the face of ongoing environmental changes.

The present study is further in line with some non-traditional approaches in both marine and terrestrial restoration concepts, highlighting the creation of entirely new “restored habitats” in regions where such habitats did not previously exist or were not documented before the dawn of early civilization. These emerging trends, such as the assisted migration concept^84–86^, are driven by the unremitting impacts of climate change that necessitate novel, as well as ecological engineering approaches^52^. Thus, the use of the RC concept in reef restoration acts and the integration of this methodology into assisted migration projects will lead to the development of well-conceived, self-perpetuating reef zones designed to maintain a variety of ecological flows, supply habitats for a wide range of reef biodiversity, and provide various ecosystem services to reef organisms and human populations alike. The results of the current experiment further present an exciting opportunity to enhance coral colonization on “bare” sand substrata, thereby promoting increased primary and secondary production, as well as augmented biodiversity.

## Methods

### Study sites

The study was conducted in the northern part of the Gulf of Eilat (Red Sea, Israel), an area subjected to extensive anthropogenic pressures including recreational activities, pollution, intermittent municipal sewage outflow, industrial installations, and the heedless development of the city of Eilat, amongst others^87,88^. The coral colonies were reared in the Eilat’s underwater floating coral nursery (29°32'28.04“N, 34°58'23.99“E), floating at 6–8 m depth (12–14 m above the seafloor), where corals were farmed under favorable conditions^34,89,90^.

The transplantation site, Kisuski Beach (29°32′48.38″N, 34°57′14.35″E), is situated 2 km southwest of the coral nursery amid a highly active recreational zone, neighboring a diving center, a water sports center, and an underwater restaurant. This site has a history of having developed patched reefs (Fig. 1a2, a2) that underwent degradation due to anthropogenic activities several decades ago. Nevertheless, even today, the area still harbors corals that manage to settle and thrive on any small available hard substrate that emerges above the sediment. The seafloor in this area is unstable, characterized by mobile sand consisting of well-sorted, medium to small grains that are constantly shifting due to currents, southern storms, and sand-dwelling organisms. As of today, hard corals are scarce at this site, with only a few isolated patches of corals, accounting for less than 10% coral coverage, and a small number of corals growing on artificial structures.

### Generating Reef Carpet (RC) units at the coral nursery

We used a total of 708 nursery-bred colonies of three branching corals, *Stylophora pistillata*, *Pocillopora damicornis*, and *Acropora* cf. *variabilis*, fragmented from adult colonies that were collected from the natural reef and from the coral nursery’s infrastructure^34,90^. The coral fragments were glued (Super Glue) on top of plastic nails and were cultured on the RC units for 8-36 months, until developing large colonial structures. Each unit consisted of a tray (a plastic net mesh 0.25 cm^2^ in size stretched over a 40 × 60 cm PVC frame) and possessed coral genotypes of a single species, along with the biota community that had developed on the tray during the nursery phase. The number of colonies per unit varied from 1 to 30 according to their sizes (5-50 cm in diameter). Ca. 20% of the colonies that became accidentally detached from the plastic nails after the preparation process were promptly reattached to the unit net using plastic cable ties. Upon the development of the coral fragments into large colonies, the RC units were placed in plastic containers filled with seawater and transferred from the nursery to the transplantation site by boat. For the present experiment, plastic-based materials were employed; however, it is anticipated that environmentally friendly alternatives will be utilized in the future development of the RC methodology.

### Reef Carpet deployment on a soft-bottom substrate

Three RC plots of 8.4m^2^ each (3 × 2.8 m) were placed 24 m to 33 m apart on the sandy seabed, at a depth of 10.7–13.3 m. Each RC comprised 194 to 294 farmed colonies assembled by joining nursery-bred RC units (35 units/RC plot) on the seabed (Fig. 1a3, 1a4; Table S1). SCUBA divers distributed the units randomly among the three RC plots and positioned them according to a predetermined scheme. This scheme maintained the planned proportion of colonies per species while ensuring a random distribution of the corals. (Fig. S1). The units in the RC plot were arranged in seven columns and five rows and were placed directly on the sand, attached to each other with plastic cable ties. Each RC was anchored to the sand using 50 cm U-shaped iron bars, fully inserted within the sediment to secure them in place.

### Monitoring

SCUBA diving surveys were conducted every 2 to 3 months for a total of 17 months starting from the initial RC deployment. During each survey, the following parameters were examined for all transplanted colonies (Table S1c; permits restricted sampling collections): (1) survival; (2) colony self-attachment: assessing the spread of tissue/skeletal growth beyond the initial attachment onto the plastic nail/net; (3) partial colony mortality (bare skeleton areas) caused by sedimentation and predation^42,43^, and/or coral senescence^91^, visually estimated in 5% intervals; (4) fish bites: Identifying lesions caused by the removal of tissue, exposing the underlying skeleton;^59^ (5) presence of the gastropod predators *Drupella cornus* and *Coralliophila sp*.; (6) disappearance from the RC. Additionally, visual censuses and underwater digital photography were used to document the development of reef-associated communities on the RCs. All fish and invertebrate specimens observed above, on, within, and between the branches of each transplanted colony were documented. Whenever possible, they were counted and identified in situ to the highest taxonomic resolution feasible. The same process was applied to each tray, including the PVC frames and plastic nets of the trays. Species identification was subsequently confirmed or modified using digital photographs and the World Register of Marine Species (WoRMS)^92^ database and by consultations with taxonomists, where applicable. Boring and within-coral biota (e.g., gall crabs, *Lithophaga* bivalves) were also included. The settlement of new coral recruits on dead coral skeletons or on the RC framework (tray or net) was visually detected and recorded at 1, 3-, and 5-months following RC transplantation.

### Planula larvae collection

The reproductive outputs of outplanted colonies of the brooding coral species *S. pistillata*^93^ were compared to naturally growing natal colonies of the same size. Planula traps^94^ were deployed during the reproductive season from sunset to sunrise on five randomly selected nights between January and August^93^. The traps were placed over 16 sexually mature ( > 8 cm diameter^95^) natal colonies and 20 randomly selected transplants from each RC. The trapped larvae were gently flushed in the laboratory using filtered seawater into wide Petri dishes and counted under a stereomicroscope^63^.

### Statistics and reproducibility

Data analysis was performed using the SPSS v.21 software for Windows. Mixed-model ANOVA was used to analyze experimental effects on the dependent variables: coral survival, disappearance, self-attachment, partial mortality, fish attacks, and the presence of gastropod predators, with time (17 months) as within-subject’s factors and coral species and between-subject’s factor. The arcsine square root transformation (for data normalization) was applied for survival, self-attachment, partial mortality, and the proportion of colonies attacked by fish and by gastropods. Mauchly’s test of sphericity was used to assess the assumption of sphericity in repeated-measures ANOVA, and Huynh-Feldt correction was used when the assumption of sphericity was violated. Tukey post-hoc tests were used to compare differences among the three coral species’ survival, self-attachment, and the proportion of colonies attacked by fish and gastropods. The Kaplan–Meier analysis was used to generate the curve of survival time for the three coral species, followed by the Mantel–Cox *log-rank* test for survival curve comparisons. Larval releases from natal and transplanted *S. pistillata* colonies were analyzed using a one-sample *t*-test, comparing natal colonies with the average of the three RCs pooled together, after the normal distribution was confirmed. To compare the diversity in each group, one-way ANOVAs were performed, followed by Tukey HSD post-hoc tests using the web-based statistical calculator resource Statpages (https://statpages.info/anova1sm.html).

In order to study community development of reef fauna on the RCs, a Canonical Correspondence Analysis (CCA) was performed with the CANOCO version 4.5^96^. We investigated the composition of reef-associated species in relation to environmental factors attributed to the coral species, the state of the coral colonies (alive/dead), time since RC transplantation, and spatial considerations (location on the RC: center or periphery; observed in/on/around corals or at the understory—i.e., on the tray/substrate underneath corals). This unimodal method of ordination allowed for visualization of the correlations between the environmental variables and the taxon in a two-dimensional ordination diagram, where the RC-associated taxa are represented by triangles, and the environmental variables are expressed by arrows outgoing from the origin (the arrow’s length reflects the importance of the variable). The statistical significance of the relationships between the species and the environmental variables was tested by the Monte Carlo permutation test, using 499 permutations under a reduced model^96^. The analysis was initially done on all the RC-related taxa, followed by a separate examination of the fish community, the invertebrate community, and the sub-group that appears to have the most intimate association with coral hosts, crustaceans (estimated as 35% of the coral-associated invertebrates^38^).

In order to explore the diversity of the developing community at the RCs, we used EstimateS version 9.1.0^97^ to calculate the non-parametric abundance-based (Chao 1) and incidence-based (Chao 2) estimators of species richness and its SD. The analysis was conducted separately for the fish fauna, the total invertebrate fauna, and the crustaceans only, based on 100 randomizations without Replacement. Chao 1 was calculated for the fish and crustacean community, whereas Chao 2 was computed for the invertebrate community (based on presence/absence data), as counting organisms under field conditions was not always possible in this group (e.g., boring organisms such as *Lithophaga* sp., colonial ascidians, etc.).

### Reporting summary

Further information on research design is available in the Nature Portfolio Reporting Summary linked to this article.

### Supplementary information

Supplementary Information

Description of Additional Supplementary Files

Supplementary Data 1

Reporting Summary

## Data Availability

All data supporting the findings of this study are available within the paper and its Supplementary Information files (including Supplementary Data 1).
