# Supplementary material for: Shifting reef restoration focus from coral survivorship to biodiversity using Reef Carpets
Source: Commun Biol. 2024 Jan 31;7:141. doi: 10.1038/s42003-024-05831-4 (PMC10830465; doi:10.1038/s42003-024-05831-4)
Supplement: Supplementary file 1 — Supplementary Information [file 42003_2024_5831_MOESM1_ESM.pdf]

Table S1.

Experimental design of three Reef Carpets deployed on a soft-bottom substrate at Kisuski Beach (Red Sea, Eilat, Israel).

(a)

	Reef Carpet 1		Reef Carpet 2		Reef Carpet 3	
	# sub-units	# colonies	# sub-units	# colonies	# sub-units	# colonies
Stylophora pistillata	10	92	11	155	11	117
Pocillopora damicornis	12	63	12	90	12	59
Acropora cf. *variabilis*	13	39	12	49	12	44
**Total**	**35**	**194**	**35**	**294**	**35**	**220**

(b)

	Distance between the Reef Carpet units (m)	
	**RC2**	**RC3**
**RC1**	29.15	33.05
**RC2**		24.05

(c)

**Monitoring type**	**Summary of activities**
Survival	Counting of whole dead colonies on the RC plots (all colonies)
Partial mortality	Bare skeleton areas caused by sedimentation/predation or coral senescence. This was performed by visually estimated in 5% intervals of the total number of dead branches in a colony.
Colony self-attachment	Assessing the spread of tissue/skeletal growth beyond the initial attachment onto the plastic nail/net (number of colonies)
Fish bites	Counting (numbers) of tissue lesions caused by fish bites, exposing the underlying skeleton
Predator gastropods	Documenting the presence and the numbers of *Drupella cornus* and *Coralliophila sp* specimens
Development of reef-associated communities	Performed by visual *in situ* censuses and analyses of underwater digital photography
Boring and within-coral biota	Gall crabs (counting each one) and *Lithophaga* bivalves (estimated numbers) were taken during field surveys
Biota- fish and invertebrates	All specimens observed above/on/within/between the branches of each colony/tray PVC frames/plastic nets / were documented. Whenever possible, they were counted and identified *in situ* to the highest possible taxonomic resolution
Biota identification	Species identification was confirmed using digital photographs and comparing with the World Register of Marine Species (WoRMS) database and by consultations with taxonomists, where applicable
Coral recruits	The settlement of new coral recruits on dead coral skeletons or on the RC framework (tray or net) was visually detected and recorded at 1, 3 and 5 months following RC transplantation.

Table S2.

List of fish taxa at the Reef Carpets over 17 months.

Phylum	Family	Genus	Species	Abbreviation
Chordata	Antennariidae	*Antennarius*	*Antennarius commerson*	**AntCom**
	Apogonidae	*Apogon*	*Apogon pselion*	**ApoPse**
	Apogonidae*	-	-	**Apogon**
	Blenniidae	*Plagiotremus*	*Plagiotremus rhinorhynchos*	**PlaRhi**
	Blenniidae	*Ecsenius*	*Ecsenius nalolo*	**EcsNal**
	Blenniidae	*Ecsenius*	*Ecsenius frontalis*	**EcsFro**
	Blenniidae	*Ecsenius*	*Ecsenius gravieri*	**EcsGra**
	Blenniidae*	-	-	**Blenni**
	Chaetodontidae	*Chaetodon*	*Chaetodon paucifasciatus*	**ChaPau**
	Chaetodontidae*	-	-	**Chaeto**
	Gobiidae	*Gobiodon*	*Gobiodon reticulatus*	**GobRet**
	Gobiidae	*Gobiodon*	*Gobiodon citrinus*	**GobCit**
	Gobiidae	*Paragobiodon*	*Paragobiodon echinocephalus*	**ParEch**
	Gobiidae	*Eviota*	-	**Eviota**
	Gobiidae	*Asterropteryx*	*Asterropteryx semipunctatus*	**AstSem**
	Gobiidae	*Istigobius*	*Istigobius decoratus*	**IstDec**
	Gobiidae	*Gnatholepis*	*Gnatholepis anjerensis*	**GnaAnj**
	Gobiidae	*Amblygobius*	*Amblygobius hectori*	**AmbHec**
	Gobiidae*	-	-	**Gobiid**
	Holocentridae	*Sargocentron*	*Sargocentron diadema*	**SarDia**
	Holocentridae	*Sargocentron*	*Sargocentron marisrubri*	**SarMar**
	Labridae	*Paracheilinus*	*Paracheilinus octotaenia*	**ParOct**
	Labridae	*Labroides*	*Labroides dimidiatus*	**LabDim**
	Labridae	*Larabicus*	*Larabicus quadrilineatus*	**LarQua**
	Labridae	*Coris*	*Coris aygula*	**CorAyg**
	Labridae	*Pseudocheilinus*	*Pseudocheilinus evanidus*	**PseEva**
	Labridae	*Pseudocheilinus*	*Pseudocheilinus hexataenia*	**PseHex**
	Labridae	*Bodianus*	*Bodianus anthoides*	**BodAnt**
	Labridae	*Cheilinus*	*Cheilinus lunulatus*	**CheLun**
	Labridae	*Thalassoma*	*Thalassoma lunare*	**ThaLun**
	Labridae*	-	-	**Labrid**
	Monacanthidae*	-	-	**Monaca**
	Muraenidae*	-	-	**Muraen**
	Ophichthidae*	-	-	**Ophich**
	Ostraciidae*	-	-	**Ostrac**
	Pomacentridae	*Dascyllus*	*Dascyllus marginatus*	**DasMar**

Pomacentridae	*Dascyllus*	*Dascyllus trimaculatus*	**DasTri**
Pomacentridae	*Dascyllus*	*Dascyllus aruanus*	**DasAru**
Pomacentridae	*Chromis*	*Chromis viridis*	**ChrVir**
Pomacentridae	*Chromis*	*Chromis flavaxilla*	**ChrFla**
Pomacentridae	*Pomacentrus*	*Pomacentrus trichourus*	**PomTri**
Pomacentridae	*Pomacentrus*	*Pomacentrus trilineatus*	**PomTri**
Pomacentridae	*Chrysiptera*	*Chrysiptera unimaculata*	**ChrUni**
Pomacentridae	*Amphiprion*	*Amphiprion bicinctus*	**AmpBic**
Pomacentridae	*Amblyglyphidodon*	*Amblyglyphidodon leucogaster*	**AmbLeu**
Pomacentridae	*Amblyglyphidodon*	-	**Amblyg**
Pseudochromidae	*Pseudochromis*	*Pseudochromis springeri*	**PseSpr**
Pseudochromidae	*Pseudochromis*	*Pseudochromis olivaceus*	**PseOli**
Pseudochromidae	*Pseudochromis*	*Pseudochromis pesi*	**PsePes**
Pseudochromidae	*Pseudochromis*	*Pseudochromis flavivertex*	**PseFla**
Pseudochromidae*	-	-	**Pseudo**
Scaridae	*Calotomus*	*Calotomus viridescens*	**CalVir**
Scaridae*	-	-	**Scarid**
Scorpaenidaae	*Scorpaenodes*	*Scorpaenodes corallinus*	**ScoCor**
Scorpaenidaae	*Sebastapistes*	*Sebastapistes cyanostigma*	**SebCya**
Scorpaenidaae	*Scorpaenopsis*	-	**Scorpa**
Scorpaenidaae	*Pterois*	-	**Pteroi**
Scorpaenidaae	*Dendrochirus*	*Dendrochirus brachypterus*	**DenBra**
Serranidae	*Pseudanthias*	-	**Pseuda**
Serranidae*	-	-	**Serran**
Sparidae	*Acanthopagrus*	*Acanthopagrus bifasciatus*	**AcaBif**
Syngnathidae	*Corythoichthys*	-	**Coryth**
Tetraodontidae	*Canthigaster*	*Canthigaster pygmaea*	**CanPyg**
Tetraodontidae	*Canthigaster*	*Canthigaster margaritata*	**CanMar**
Tetraodontidae	*Torquigener*	*Torquigener flavimaculosus*	**TorFla**
FishUnidentified	-	-	**FishUn**

*****= lowest taxonomic rank identified when not recognized to genus or species level.

Table S3. List of invertebrate taxa at the Reef Carpets over 17 months.

Phylum	Subphylum	Class	Subclass	Order	Infraorder	Family	Genus	Species	Abbreviation
Annelida	NR	Polychaeta	Sedentaria	Sabellida	NR	Serpulidae	*Spirobranchus*	-	**Spirob**
	NR	Polychaeta	Sedentaria	Sabellida	NR	Serpulidae*	-	-	**Serpul**
	NR	Polychaeta	Sedentaria	Sabellida	NR	Sabellidae*	-	-	**Sabell**
	NR	Polychaeta	Sedentaria	Terebellida	NR	Terebellidae*	-	-	**Terebe**
	NR	Polychaeta	Errantia	Phyllodocida*	-	-	-	-	**Phyllo**
Arthropoda	Crustacea	Malacostraca	Eumalacostraca	Decapoda	Brachyura	Trapeziidae	*Trapezia*	*Trapezia cymodoce*	**TraCym**
	Crustacea	Malacostraca	Eumalacostraca	Decapoda	Brachyura	Trapeziidae	*Trapezia*	*Trapezia tigrina*	**TraTig**
	Crustacea	Malacostraca	Eumalacostraca	Decapoda	Brachyura	Trapeziidae	*Trapezia*	*Trapezia digitalis*	**TraDig**
	Crustacea	Malacostraca	Eumalacostraca	Decapoda	Brachyura	Trapeziidae	*Trapezia*	*Trapezia guttata*	**TraGut**
	Crustacea	Malacostraca	Eumalacostraca	Decapoda	Brachyura	Tetraliidae	*Tetraloides*	*Tetraloides nigrifrons*	**TetNig**
	Crustacea	Malacostraca	Eumalacostraca	Decapoda	Brachyura	Tetraliidae	*Tetralia*	*Tetralia cavimana*	**TetCav**
	Crustacea	Malacostraca	Eumalacostraca	Decapoda	Brachyura	Xanthidae	*Cymo*	*Cymo andreossyi*	**CymAnd**
	Crustacea	Malacostraca	Eumalacostraca	Decapoda	Brachyura	Cryptochiridae	*Hapalocarcinus*	*Hapalocarcinus marsupialis*	**HapMar**
	Crustacea	Malacostraca	Eumalacostraca	Decapoda	Brachyura	Majoidea*	-	-	**Majoid**
	Crustacea	Malacostraca	Eumalacostraca	Decapoda	Brachyura	Portunidae*	-	-	**Portun**
	Crustacea	Malacostraca	Eumalacostraca	Decapoda	Brachyura1*	-	-	-	**Brac1**
	Crustacea	Malacostraca	Eumalacostraca	Decapoda	Brachyura2*	-	-	-	**Brac2**
	Crustacea	Malacostraca	Eumalacostraca	Decapoda	Brachyura3*	-	-	-	**Brac3**
	Crustacea	Malacostraca	Eumalacostraca	Decapoda	Brachyura4*	-	-	-	**Brac4**
	Crustacea	Malacostraca	Eumalacostraca	Decapoda	Brachyura5*	-	-	-	**Brac5**
	Crustacea	Malacostraca	Eumalacostraca	Decapoda	Brachyura6*	-	-	-	**Brac6**

	Crustacea	Malacostraca	Eumalacostraca	Decapoda	Brachyura7*	-	-	-	**Brac7**
	Crustacea	Malacostraca	Eumalacostraca	Decapoda	Brachyura8*	-	-	-	**Brac8**
	Crustacea	Malacostraca	Eumalacostraca	Decapoda	Brachyura9*	-	-	-	**Brac9**
	Crustacea	Malacostraca	Eumalacostraca	Decapoda	Brachyura10*	-	-	-	**Brac10**
	Crustacea	Malacostraca	Eumalacostraca	Decapoda	Brachyura11*	-	-	-	**Brac11**
	Crustacea	Malacostraca	Eumalacostraca	Decapoda	Caridea	Alpheidae	*Alpheus*	*Alpheus lottini*	**AlpLot**
	Crustacea	Malacostraca	Eumalacostraca	Decapoda	Caridea	Palaemonidae	*Periclimenes*	-	**Pericl**
	Crustacea	Malacostraca	Eumalacostraca	Decapoda	Caridea	Palaemonidae	*Urocaridella*	*Urocaridella antonbruuni*	**UroAnt**
	Crustacea	Malacostraca	Eumalacostraca	Decapoda	Caridea	Stenopodidae	*Stenopus*	*Stenopus hispidus*	**SteHis**
	Crustacea	Malacostraca	Eumalacostraca	Decapoda	Caridea	Hippolytidae	*Saron*	*Saron marmoratus*	**SarMar**
	Crustacea	Malacostraca	Eumalacostraca	Decapoda	Caridea	Thoridae	*Thor*	*Thor amboinensis*	**ThoAmb**
	Crustacea	Malacostraca	Eumalacostraca	Decapoda	Caridea	Lysmatidae	*Lysmata*	*Lysmata amboinensis*	**LysAmb**
	Crustacea	Malacostraca	Eumalacostraca	Decapoda	Caridea1*	-	-	-	**Carid1**
	Crustacea	Malacostraca	Eumalacostraca	Decapoda	Caridea2*	-	-	-	**Carid2**
	Crustacea	Malacostraca	Eumalacostraca	Decapoda	Caridea3*	-	-	-	**Carid3**
	Crustacea	Malacostraca	Eumalacostraca	Decapoda	Caridea4*	-	-	-	**Carid4**
	Crustacea	Malacostraca	Eumalacostraca	Decapoda	Caridea5*	-	-	-	**Carid5**
	Crustacea	Malacostraca	Eumalacostraca	Decapoda	Caridea6*	-	-	-	**Carid6**
	Crustacea	Malacostraca	Eumalacostraca	Decapoda	Caridea*	-	-	-	**Carid**
	Crustacea	Malacostraca	Eumalacostraca	Decapoda	Anomura	Paguroidea*	-	-	**Paguro**
	Crustacea	Malacostraca	Eumalacostraca	Decapoda	Anomura	Galatheidae	*Galathea*	-	**Galath**
	Crustacea	Malacostraca	Eumalacostraca	Decapoda*	-	-	-	-	**Decapo**
	Crustacea	Malacostraca	Hoplocarida	Stomatopoda*	-	-	-	-	**Stomat**
Bryozoa	NR	Gymnolaemata	NR	Cheilostomatida	NR	Bugulidae	*Bugula*	-	**Bugula**

Chordata	Tunicata	Ascidiacea	NR	Phlebobranchia	NR	Ascidiidae	*Phallusia*	*Phallusia nigra*	**PhaNig**
	Tunicata	Ascidiacea	NR	Phlebobranchia	NR	Ascidiidae	*Phallusia*	*Phallusia arabica*	**PhaAra**
	Tunicata	Ascidiacea	NR	Phlebobranchia	NR	Corellidae	*Rhodosoma*	*Rhodosoma turcicum*	**RhoTur**
	Tunicata	Ascidiacea	NR	Stolidobranchia	NR	Pyuridae	*Halocynthia*	*Halocynthia spinosa*	**HalSpi**
	Tunicata	Ascidiacea	NR	Stolidobranchia	NR	Pyuridae	*Pyura*	*Pyura gangelion*	**PyuGan**
	Tunicata	Ascidiacea	NR	Stolidobranchia	NR	Pyuridae	*Boltenia*	*Boltenia yossiloya*	**BolYos**
	Tunicata	Ascidiacea	NR	Stolidobranchia	NR	Pyuridae	*Herdmania*	*Herdmania momus*	**HerMom**
	Tunicata	Ascidiacea	NR	Stolidobranchia	NR	Styelidae	*Botryllus*	*Botryllus eilatensis*	**BotEil**
	Tunicata	Ascidiacea	NR	Stolidobranchia	NR	Styelidae	*Polycarpa*	*Polycarpa cryptocarpa*	**PolCry**
	Tunicata	Ascidiacea	NR	Stolidobranchia	NR	Styelidae	*Polycarpa*	*Polycarpa mytiligera*	**PolMyt**
	Tunicata	Ascidiacea	NR	Aplousobranchia	NR	Diazonidae	*Rhopalaea*	-	**Rhopal**
	Tunicata	Ascidiacea	NR	Aplousobranchia	NR	Didemnidae	*Diplosoma*	-	**Diplos**
	Tunicata	Ascidiacea	NR	Aplousobranchia	NR	Didemnidae	*Didemnum*	-	**Didemn**
	Tunicata	Ascidiacea1*	-	-	-	-	-	-	**Ascid1**
	Tunicata	Ascidiacea*	-	-	-	-	-	-	**Ascidi**
Cnidaria	NR	Anthozoa	Hexacorallia	Actiniaria*	-	-	-	-	**Actini**
	NR	Anthozoa	Octocorallia	Alcyonacea	NR	Xeniidae*	-	-	**Xeniid**
	NR	Scyphozoa	Discomedusae	Rhizostomeae	NR	Cassiopeidae	*Cassiopea*	*Cassiopea andromeda*	**CasAnd**
Echinodermata	Echinozoa	Echinoidea	Euechinoidea	Diadematoida	NR	Diadematidae	*Diadema*	*Diadema setosum*	**DiaSet**
	Echinozoa	Echinoidea	Euechinoidea	Diadematoida	NR	Diadematidae	*Echinothrix*	*Echinothrix calamaris*	**EchCal**
	Echinozoa	Echinoidea	Euechinoidea	Echinothurioida	NR	Echinothuriidae	*Asthenosoma*	*Asthenosoma marisrubri*	**AstMar**
	Echinozoa	Echinoidea	Euechinoidea	Camarodonta	Echinidea	Echinometridae	*Echinometra*	*Echinometra mathaei*	**EchMat**
	Echinozoa	Echinoidea	Euechinoidea	Camarodonta	Echinidea	Echinometridae	*Heterocentrotus*	*Heterocentrotus mamillatus*	**HetMam**
	Echinozoa	Echinoidea	Euechinoidea	Camarodonta	Echinidea	Echinometridae	*Echinostrephus*	*Echinostrephus molaris*	**EchMol**

	Echinozoa	Echinoidea	Euechinoidea	Camarodonta	Echinidea	Toxopneustidae	*Tripneustes*	*Tripneustes gratilla elatensis*	**TriGra**
	Echinozoa	Echinoidea	Cidaroidea	Cidaroida	NR	Cidaridae*	-	-	**Cidari**
	Asterozoa	Ophiuroidea	Myophiuroidea	Amphilepidida	NR	Ophiotrichidae	*Ophiothrix*	-	**Ophiot**
	Asterozoa	Ophiuroidea	Myophiuroidea	Ophiacanthida	NR	Ophiocomidae	*Ophiocoma*	-	**Ophioc**
	Asterozoa	Ophiuroidea	Myophiuroidea	Ophiurida*	-	-	-	-	**Ophiu1**
	Asterozoa	Asteroidea*	-	-	-	-	-	-	**Astero**
	Echinozoa	Holothuroidea*	-	-	-	-	-	-	**Holoth**
	Crinozoa	Crinoidea*	-	-	-	-	-	-	**Crinoi**
Foraminifera	NR	Tubothalamea	NR	Miliolida	NR	Soritidae*	-	-	**Soriti**
Mollusca	NR	Bivalvia	Pteriomorphia	Mytilida	NR	Mytilidae	*Lithophaga*†	-	**Lithop**
	NR	Bivalvia	Pteriomorphia	Mytilida	NR	Mytilidae*	-	-	**Mytili**
	NR	Bivalvia	Pteriomorphia	Ostreida	NR	Pteriidae	*Pteria*	-	**Pteria**
	NR	Bivalvia	Pteriomorphia	Ostreida	NR	Pteriidae	*Pinctada*	-	**Pincta**
	NR	Bivalvia	Pteriomorphia	Ostreida	NR	Pinnidae	*Streptopinna*	*Streptopinna saccata*	**StrSac**
	NR	Bivalvia	Pteriomorphia	Ostreida	NR	Pinnidae	*Pinna*	*Pinna muricata*	**PinMur**
	NR	Bivalvia	Pteriomorphia	Ostreida	NR	Ostreidae1*	-	-	**Ostre1**
	NR	Bivalvia	Pteriomorphia	Ostreida	NR	Ostreidae2*	-	-	**Ostre2**
	NR	Bivalvia	Pteriomorphia	Ostreida	NR	Ostreidae3*	-	-	**Ostre3**
	NR	Bivalvia	Pteriomorphia	Arcida	NR	Arcidae*	-	-	**Arcida**
	NR	Bivalvia	Pteriomorphia	Pectinida	NR	Pectinidae*	-	-	**Pectin**
	NR	Bivalvia	Heterodonta	Cardiida	NR	Cardiidae (Subfamily Tridacnidae*)	-	-	**Tridac**
	NR	Bivalvia1*	-	-	-	-	-	-	**Bival1**
	NR	Bivalvia*	-	-	-	-	-	-	**Bivalv**
	NR	Gastropoda	Caenogastropoda	Littorinimorpha	NR	Cypraeidae*	-	-	**Cyprae**

	NR	Gastropoda	Caenogastropoda	Littorinimorpha	NR	Vermetidae	*Serpulorbis*	*Serpulorbis inopertus*	**SerIno**
	NR	Gastropoda	Caenogastropoda	Neogastropoda	NR	Muricidae	*Drupella*	*Drupella cornus*	**DruCor**
	NR	Gastropoda	Caenogastropoda	Neogastropoda	NR	Muricidae	*Coralliophila*	-	**Corall**
	NR	Gastropoda	Caenogastropoda	Neogastropoda	NR	Muricidae*	-	-	**Murici**
	NR	Gastropoda	Caenogastropoda	Neogastropoda	NR	Conidae	*Conus*	-	**Conus**
	NR	Gastropoda	Caenogastropoda	Neogastropoda	NR	Nassariidae*	-	-	**Nassar**
	NR	Gastropoda	Patellogastropoda*	-	-	-	-	-	**Patell**
	NR	Gastropoda	Heterobranchia	Nudibranchia*	-	-	-	-	**Nudibr**
	NR	Gastropoda*	-	-	-	-	-	-	**Gastro**
Porifera	NR	Demospongiae	Heteroscleromorpha	Poecilosclerida	NR	Podospongiidae	*Negombata*	*Negombata magnifica*	**NegMag**
	NR	Demospongiae	Heteroscleromorpha	Poecilosclerida	NR	Mycalidae	*Mycale*	*Mycale fistulifera*	**MycFis**
	NR	Demospongiae	Heteroscleromorpha	Clionaida	NR	Clionaidae*	-	-	**Clioni**
	NR	Demospongiae	Heteroscleromorpha	Clionaida	NR	Clionaidae	*Cliona*	*Cliona viridis*	**CliVir**
	NR	Calcarea	Calcaronea	Leucosolenida	NR	Grantiidae	*Grantia*	-	**Granti**
Porifera1*	-	-	-	-	-	-	-	-	**Pori1**
Porifera2*	-	-	-	-	-	-	-	-	**Pori2**
Porifera3*	-	-	-	-	-	-	-	-	**Pori3**
Porifera4*	-	-	-	-	-	-	-	-	**Pori4**
Porifera5*	-	-	-	-	-	-	-	-	**Pori5**
Porifera6*	-	-	-	-	-	-	-	-	**Pori6**
Porifera7*	-	-	-	-	-	-	-	-	**Pori7**
Porifera8*	-	-	-	-	-	-	-	-	**Pori8**
Porifera9*	-	-	-	-	-	-	-	-	**Pori9**
Porifera10*	-	-	-	-	-	-	-	-	**Pori10**

Porifera11*	-	-	-	-	-	-	-	-	**Pori11**
Porifera12*	-	-	-	-	-	-	-	-	**Pori12**
Porifera13*	-	-	-	-	-	-	-	-	**Pori13**
Porifera14*	-	-	-	-	-	-	-	-	**Pori14**
Porifera*	-	-	-	-	-	-	-	-	**Porife**
Xenacoelomorpha	Acoelomorpha	NR	NR	Acoela	Crucimusculata	Convolutidae	*Waminoa*	*Waminoa brickneri*	**WamBri**

*****= lowest taxonomic rank identified when not recognized to genus or species level.

NR= No rank

† The boring bivalves commonly associated with *S. pistillata* colonies in Eilat are *Leiosolenus lessepsianus* (formerly *Lithophaga lessepsiana*). However, due to the difficulty to validate the taxonomy of boring species under field conditions, we assigned all boring *Lithophaga/Leiosolenus* species to *Lithophaga* genus.

Table S4. Statistical comparison of mean survival times, estimated by the Kaplan-Meier analysis, for the three coral species transplanted as Reef Carpets during 17 months post-transplantation.

	**Median (months)**			
	**Estimate**	**Std. Error**	**95% Confidence Interval (months)**	
			**Lower Bound**	**Upper Bound**
Acropora cf. *variabilis*	17	0.221	16.567	17.433
Pocillopora damicornis	13	0.535	11.952	14.048
Stylophora pistillata	11	0.514	9.992	12.008

	**Chi-Square**	***df***	**P**
Log Rank (Mantel-Cox)	7.087	2	0.029

Fish

Table S5. Results of ANOVA test, followed by Tukey HSD Post-hoc tests, of the fish diversity recorded in the 3 Reef Carpet coral species, *A.* cf. *variabilis*, *P. damicornis* and *S. pistillata.*

Source of Variation	Sum of Squares	*df*	Variance	F	p
Between Groups	262509.3	2	131254.6	271.7107	<0.001
Within Groups	2786334	5768	483.0676		
**Total**	**3048843**	**5770**			

**Tukey HSD Post-hoc Tests**				
	**Mean difference**	**95% confidence interval**		**p**
		**Lower bound**	**Upper bound**	
A. cf. *variabilis* vs. *P. damicornis*	-6.3800	-8.3227	-4.4373	<0.001
A. cf. *variabilis* vs. *S. pistillata*	8.9800	7.2067	10.7533	<0.001
P. damicornis vs. *S. pistillata*	15.3600	13.7856	16.9344	<0.001

Table S6: Results of ANOVA test, followed by Tukey HSD Post-hoc tests, of the fish diversity recorded in live colonies, dead colonies, and on the substrate of *A.* cf. *variabilis*, *P. damicornis* and *S. pistillata* composing the 3 Reef Carpets.

Source of Variation	Sum of Squares	*df*	Variance	F	p
Between Groups	1559540	8	194942.5	853.4352	<0.001
Within Groups	1316161	5762	228.4209		
**Total**	**2875701**	**5770**			

**Tukey HSD Post-hoc Tests**				
	**Mean difference**	**95% confidence interval**		**p**
		**Lower bound**	**Upper bound**	
A. cf. *variabilis* Alive vs. *A.* cf. *variabilis* Dead	-67.2700	-72.6307	-61.9093	<0.001
A. cf. *variabilis* Alive vs. *A.* cf. *variabilis* Tray	-34.4500	-37.7761	-31.1239	<0.001
A. cf. *variabilis* Alive vs. *P. damicornis* Alive	-36.1000	-38.2265	-33.9735	<0.001
A. cf. *variabilis* Alive vs. *P. damicornis* Dead	-53.7300	-56.7895	-50.6705	<0.001
A. cf. *variabilis* Alive vs. *P. damicornis* Tray	-15.6100	-18.9713	-12.2487	<0.001
A. cf. *variabilis* Alive vs. *S. pistillata* Alive	-37.5000	-39.4245	-35.5755	<0.001
A. cf. *variabilis* Alive vs. *S. pistillata* Dead	-52.2600	-54.6588	-49.8612	<0.001

A. cf. *variabilis* Alive vs. *S. pistillata* Tray	-26.1800	-29.7060	-22.6540	<0.001
A. cf. *variabilis* Dead vs. *A.* cf. *variabilis* Tray	32.8200	26.9342	38.7058	<0.001
A. cf. *variabilis* Dead vs. *P. damicornis* Alive	31.1700	25.8689	36.4711	<0.001
A. cf. *variabilis* Dead vs. *P. damicornis* Dead	13.5400	7.8006	19.2794	<0.001
A. cf. *variabilis* Dead vs. *P. damicornis* Tray	51.6600	45.7542	57.5658	<0.001
A. cf. *variabilis* Dead vs. *S. pistillata* Alive	29.7700	24.5467	34.9933	<0.001
A. cf. *variabilis* Dead vs. *S. pistillata* Dead	15.0100	9.5939	20.4261	<0.001
A. cf. *variabilis* Dead vs. *S. pistillata* Tray	41.0900	35.0890	47.0910	<0.001
A. cf. *variabilis* Tray vs. *P. damicornis* Alive	-1.6500	-4.8790	1.5790	0.8133
A. cf. *variabilis* Tray vs. *P. damicornis* Dead	-19.2800	-23.1871	-15.3729	<0.001
A. cf. *variabilis* Tray vs. *P. damicornis* Tray	18.8400	14.6923	22.9877	<0.001
A. cf. *variabilis* Tray vs. *S. pistillata* Alive	-3.0500	-6.1497	0.0497	0.0579
A. cf. *variabilis* Tray vs. *S. pistillata* Dead	-17.8100	-21.2245	-14.3955	<0.001
A. cf. *variabilis* Tray vs. *S. pistillata* Tray	8.2700	3.9878	12.5522	<0.001
P. damicornis Alive vs. *P. damicornis* Dead	-17.6300	-20.5837	-14.6763	<0.001
P. damicornis Alive vs. *P. damicornis* Tray	20.4900	17.2247	23.7553	<0.001
P. damicornis Alive vs. *S. pistillata* Alive	-1.4000	-3.1514	0.3514	0.2422
P. damicornis Alive vs. *S. pistillata* Dead	-16.1600	-18.4223	-13.8977	<0.001
P. damicornis Alive vs. *S. pistillata* Tray	9.9200	6.4854	13.3546	<0.001
P. damicornis Dead vs. *P. damicornis* Tray	38.1200	34.1828	42.0572	<0.001
P. damicornis Dead vs. *S. pistillata* Alive	16.2300	13.4182	19.0418	<0.001
P. damicornis Dead vs. *S. pistillata* Dead	1.4700	-1.6855	4.6255	0.8803
P. damicornis Dead vs. *S. pistillata* Tray	27.5500	23.4714	31.6286	<0.001
P. damicornis Tray vs. *S. pistillata* Alive	-21.8900	-25.0275	-18.7525	<0.001
P. damicornis Tray vs. *S. pistillata* Dead	-36.6500	-40.0989	-33.2011	<0.001
P. damicornis Tray vs. *S. pistillata* Tray	-10.5700	-14.8796	-6.2604	<0.001
S. pistillata Alive vs. *S. pistillata* Dead	-14.7600	-16.8336	-12.6864	<0.001
S. pistillata Alive vs. *S. pistillata* Tray	11.3200	8.0067	14.6333	<0.001
S. pistillata Dead vs. *S. pistillata* Tray	26.0800	22.4705	29.6895	<0.001

<u>**Invertebrates**</u>

Table S7. Results of ANOVA test, followed by Tukey HSD Post-hoc tests, of the invertebrate diversity recorded in the 3 Reef Carpet coral species, *A.* cf. *variabilis*, *P. damicornis* and *S. pistillata.*

Source of Variation	Sum of Squares	*df*	Variance	F	p
Between Groups	305715.4	2	152857.7	2885.123	<0.001
Within Groups	305596.4	5768	52.9813		
**Total**	**611311.8**	**5770**			

Tukey HSD Post-hoc Tests

	Mean difference	95% confidence interval		p
		Lower bound	Upper bound	
A. cf. *variabilis* vs. *P. damicornis*	20.3900	19.7466	21.0334	<0.001
A. cf. *variabilis* vs. *S. pistillata*	15.0500	14.4627	15.6373	<0.001
P. damicornis vs. *S. pistillata*	-5.3400	-5.8614	-4.8186	<0.001

Table S8. Results of ANOVA test, followed by Tukey HSD Post-hoc tests, of the invertebrate diversity recorded in live colonies, dead colonies, and on the substrate of *A.* cf. *variabilis*, *P. damicornis* and *S. pistillata* composing the 3 Reef Carpets.

Source of Variation	Sum of Squares	*df*	Variance	F	p
Between Groups	1299313	8	162414.1	4302.382	<0.001
Within Groups	217514.5	5762	37.7498		
**Total**	**1516827**	**5770**			

Tukey HSD Post-hoc Tests

	Mean difference	95% confidence interval		p
		Lower bound	Upper	
A. cf. *variabilis* Alive vs. *A.* cf. *variabilis* Dead	-41.0800	-43.2593	-38.9007	<0.001
A. cf. *variabilis* Alive vs. *A.* cf. *variabilis* Tray	-44.2800	-45.6321	-42.9279	<0.001
A. cf. *variabilis* Alive vs. *P. damicornis* Alive	2.0500	1.1855	2.9145	<0.001
A. cf. *variabilis* Alive vs. *P. damicornis* Dead	-19.5700	-20.8138	-18.3262	<0.001
A. cf. *variabilis* Alive vs. *P. damicornis* Tray	-18.5100	-19.8765	-17.1435	<0.001
A. cf. *variabilis* Alive vs. *S. pistillata* Alive	4.7900	4.0077	5.5723	<0.001
A. cf. *variabilis* Alive vs. *S. pistillata* Dead	-10.2000	-11.1752	-9.2248	<0.001
A. cf. *variabilis* Alive vs. *S. pistillata* Tray	-47.0100	-48.4434	-45.5766	<0.001
A. cf. *variabilis* Dead vs. *A.* cf. *variabilis* Tray	-3.2000	-5.5927	-0.8073	0.001
A. cf. *variabilis* Dead vs. *P. damicornis* Alive	43.1300	40.9750	45.2850	<0.001
A. cf. *variabilis* Dead vs. *P. damicornis* Dead	21.5100	19.1768	23.8432	<0.001
A. cf. *variabilis* Dead vs. *P. damicornis* Tray	22.5700	20.1692	24.9708	<0.001
A. cf. *variabilis* Dead vs. *S. pistillata* Alive	45.8700	43.7466	47.9934	<0.001
A. cf. *variabilis* Dead vs. *S. pistillata* Dead	30.8800	28.6782	33.0818	<0.001
A. cf. *variabilis* Dead vs. *S. pistillata* Tray	-5.9300	-8.3696	-3.4904	<0.001
A. cf. *variabilis* Tray vs. *P. damicornis* Alive	46.3300	45.0173	47.6427	<0.001
A. cf. *variabilis* Tray vs. *P. damicornis* Dead	24.7100	23.1216	26.2984	<0.001
A. cf. *variabilis* Tray vs. *P. damicornis* Tray	25.7700	24.0839	27.4561	<0.001
A. cf. *variabilis* Tray vs. *S. pistillata* Alive	49.0700	47.8099	50.3301	<0.001
A. cf. *variabilis* Tray vs. *S. pistillata* Dead	34.0800	32.6919	35.4681	<0.001
A. cf. *variabilis* Tray vs. *S. pistillata* Tray	-2.7300	-4.4708	-0.9892	<0.001
P. damicornis Alive vs. *P. damicornis* Dead	-21.6200	-22.8208	-20.4192	<0.001
P. damicornis Alive vs. *P. damicornis* Tray	-20.5600	-21.8874	-19.2326	<0.001
P. damicornis Alive vs. *S. pistillata* Alive	2.7400	2.0280	3.4520	<0.001
P. damicornis Alive vs. *S. pistillata* Dead	-12.2500	-13.1697	-11.3303	<0.001
P. damicornis Alive vs. *S. pistillata* Tray	-49.0600	-50.4562	-47.6638	<0.001

P. damicornis Dead vs. *P. damicornis* Tray	1.0600	-0.5406	2.6606	0.5053
P. damicornis Dead vs. *S. pistillata* Alive	24.3600	23.2169	25.5031	<0.001
P. damicornis Dead vs. *S. pistillata* Dead	9.3700	8.0872	10.6528	<0.001
P. damicornis Dead vs. *S. pistillata* Tray	-27.4400	-29.0981	-25.7819	<0.001
P. damicornis Tray vs. *S. pistillata* Alive	23.3000	22.0245	24.5755	<0.001
P. damicornis Tray vs. *S. pistillata* Dead	8.3100	6.9079	9.7121	<0.001
P. damicornis Tray vs. *S. pistillata* Tray	-28.5000	-30.2520	-26.7480	<0.001
S. pistillata Alive vs. *S. pistillata* Dead	-14.9900	-15.8330	-14.1470	<0.001
S. pistillata Alive vs. *S. pistillata* Tray	-51.8000	-53.1469	-50.4531	<0.001
S. pistillata Dead vs. *S. pistillata* Tray	-36.8100	-38.2774	-35.3426	<0.001

Crustaceans

Table S9. Results of ANOVA test, followed by Tukey HSD Post-hoc tests, of the crustacean diversity recorded in the 3 Reef Carpet coral species, *A.* cf. *variabilis*, *P. damicornis* and *S. pistillata*.

Source of Variation	Sum of Squares	*df*	Variance	F	p
Between Groups	21079.05	2	10539.52	3665.725	<0.001
Within Groups	16583.89	5768	2.8752		
**Total**	**37662.94**	**5770**			

Tukey HSD Post-hoc Tests

	Mean difference	95% confidence interval		p
		Lower bound	Upper bound	
A. cf. *variabilis* vs. *P. damicornis*	5.0000	4.8501	5.1499	<0.001
A. cf. *variabilis* vs. *S. pistillata*	4.5000	4.3632	4.6368	<0.001
P. damicornis vs. *S. pistillata*	-0.5000	-0.6215	-0.3785	<0.001

Table S10. Results of ANOVA test, followed by Tukey HSD Post-hoc tests, of the crustacean diversity recorded in live colonies, dead colonies, and on the substrate of *A.* cf. *variabilis*, *P. damicornis* and *S. pistillata* composing the 3 Reef Carpets.

Source of Variation	Sum of Squares	*df*	Variance	F	p
Between Groups	523877	8	65484.63	4571.741	<0.001
Within Groups	82533.64	5762	14.3238		
**Total**	**606410.7**	**5770**			

Tukey HSD Post-hoc Tests

	Mean difference	95% confidence interval		p
		Lower bound	Upper bound	
A. cf. *variabilis* Alive vs. *A.* cf. *variabilis* Dead	-33.5300	-34.8724	-32.1876	<0.001
A. cf. *variabilis* Alive vs. *A.* cf. *variabilis* Tray	-30.0000	-30.8329	-29.1671	<0.001
A. cf. *variabilis* Alive vs. *P. damicornis* Alive	-1.7500	-2.2825	-1.2175	<0.001
A. cf. *variabilis* Alive vs. *P. damicornis* Dead	-7.5400	-8.3062	-6.7738	<0.001
A. cf. *variabilis* Alive vs. *P. damicornis* Tray	-20.3300	-21.1717	-19.4883	<0.001
A. cf. *variabilis* Alive vs. *S. pistillata* Alive	-2.0000	-2.4819	-1.5181	<0.001
A. cf. *variabilis* Alive vs. *S. pistillata* Dead	-14.2200	-14.8207	-13.6193	<0.001
A. cf. *variabilis* Alive vs. *S. pistillata* Tray	-30.6800	-31.5630	-29.7970	<0.001
A. cf. *variabilis* Dead vs. *A.* cf. *variabilis* Tray	3.5300	2.0561	5.0039	<0.001
A. cf. *variabilis* Dead vs. *P. damicornis* Alive	31.7800	30.4525	33.1075	<0.001
A. cf. *variabilis* Dead vs. *P. damicornis* Dead	25.9900	24.5528	27.4272	<0.001
A. cf. *variabilis* Dead vs. *P. damicornis* Tray	13.2000	11.7211	14.6789	<0.001
A. cf. *variabilis* Dead vs. *S. pistillata* Alive	31.5300	30.2220	32.8380	<0.001
A. cf. *variabilis* Dead vs. *S. pistillata* Dead	19.3100	17.9537	20.6663	<0.001
A. cf. *variabilis* Dead vs. *S. pistillata* Tray	2.8500	1.3473	4.3527	<0.001
A. cf. *variabilis* Tray vs. *P. damicornis* Alive	28.2500	27.4414	29.0586	<0.001
A. cf. *variabilis* Tray vs. *P. damicornis* Dead	22.4600	21.4816	23.4384	<0.001
A. cf. *variabilis* Tray vs. *P. damicornis* Tray	9.6700	8.6314	10.7086	<0.001
A. cf. *variabilis* Tray vs. *S. pistillata* Alive	28.0000	27.2238	28.7762	<0.001
A. cf. *variabilis* Tray vs. *S. pistillata* Dead	15.7800	14.9249	16.6351	<0.001
A. cf. *variabilis* Tray vs. *S. pistillata* Tray	-0.6800	-1.7523	0.3923	0.5668
P. damicornis Alive vs. *P. damicornis* Dead	-5.7900	-6.5297	-5.0503	<0.001
P. damicornis Alive vs. *P. damicornis* Tray	-18.5800	-19.3977	-17.7623	<0.001
P. damicornis Alive vs. *S. pistillata* Alive	-0.2500	-0.6886	0.1886	0.7034

P. damicornis Alive vs. *S. pistillata* Dead	-12.4700	-13.0365	-11.9035	<0.001
P. damicornis Alive vs. *S. pistillata* Tray	-28.9300	-29.7901	-28.0699	<0.001
P. damicornis Dead vs. *P. damicornis* Tray	-12.7900	-13.7759	-11.8041	<0.001
P. damicornis Dead vs. *S. pistillata* Alive	5.5400	4.8359	6.2441	<0.001
P. damicornis Dead vs. *S. pistillata* Dead	-6.6800	-7.4702	-5.8898	<0.001
P. damicornis Dead vs. *S. pistillata* Tray	-23.1400	-24.1614	-22.1186	<0.001
P. damicornis Tray vs. *S. pistillata* Alive	18.3300	17.5443	19.1157	<0.001
P. damicornis Tray vs. *S. pistillata* Dead	6.1100	5.2463	6.9737	<0.001
P. damicornis Tray vs. *S. pistillata* Tray	-10.3500	-11.4292	-9.2708	<0.001
S. pistillata Alive vs. *S. pistillata* Dead	-12.2200	-12.7393	-11.7007	<0.001
S. pistillata Alive vs. *S. pistillata* Tray	-28.6800	-29.5097	-27.8503	<0.001
S. pistillata Dead vs. *S. pistillata* Tray	-16.4600	-17.3639	-15.5561	<0.001

Table S11. New coral settlement recorded in Reef Carpet (RC) units of the three RC coral species, *A.* cf. *variabilis*, *P. damicornis* and *S. pistillata*, at 1, 3 and 5 months following RC assemblage. At each of the three RC plots (RC1-RC3), the number of units with new coral settlement (out of 35 units comprising a RC plot) is presented. New recruits were spotted either on skeletons of dead RC-corals, or on the RC framework (i.e., tray or net).

RC 1

Month post RC transplantation	Coral species of RC unit	Number of RC units with new settlements (n=35)	Number of new coral recruits	Recruits seen on:	
				RC coral skeletons	RC framework
1	*A.* cf. *variabilis*	4	11	0	11
	S. pistillata	4	11	3	8
	P. damicornis	1	6	0	6
	**Total**	**9**	**28**	**3**	**25**
3	*A.* cf. *variabilis*	6	26	2	24
	S. pistillata	7	25	5	20
	P. damicornis	3	4	3	1
	**Total**	**16**	**55**	**10**	**45**
5	*A.* cf. *variabilis*	4	7	2	5
	S. pistillata	8	15	7	8
	P. damicornis	1	1	1	0
	**Total**	**13**	**23**	**10**	**13**

RC 2

Month post RC transplantation	Coral species of RC unit	Number of RC units with new settlements	Number of new coral recruits	Recruits seen on:	
				RC coral skeletons	RC framework
1	*A.* cf. *variabilis*	2	9	1	8
	S. pistillata	3	8	1	7
	P. damicornis	4	5	3	2
	**Total**	**9**	**22**	**5**	**17**
3	*A.* cf. *variabilis*	4	7	4	3
	S. pistillata	10	17	5	12
	P. damicornis	5	7	6	1
	**Total**	**19**	**31**	**15**	**16**
5	*A.* cf. *variabilis*	2	6	2	4
	S. pistillata	7	16	11	5
	P. damicornis	5	6	6	0
	**Total**	**14**	**28**	**19**	**9**

RC 3					
Month post RC transplantation	Coral species of RC unit	Number of RC units with new settlements	Number of new coral recruits	Recruits seen on:	
				RC coral skeletons	RC framework
1	*A.* cf. *variabilis*	7	18	1	17
	S. pistillata	9	53	7	46
	P. damicornis	4	16	1	15
	**Total**	**20**	**87**	**9**	**78**
3	*A.* cf. *variabilis*	4	8	0	8
	S. pistillata	5	9	4	5
	P. damicornis	2	8	1	7
	**Total**	**11**	**25**	**5**	**20**
5	*A.* cf. *variabilis*	1	1	0	1
	S. pistillata	5	14	10	4
	P. damicornis	5	7	5	2
	**Total**	**11**	**22**	**15**	**7**

Fig. S1

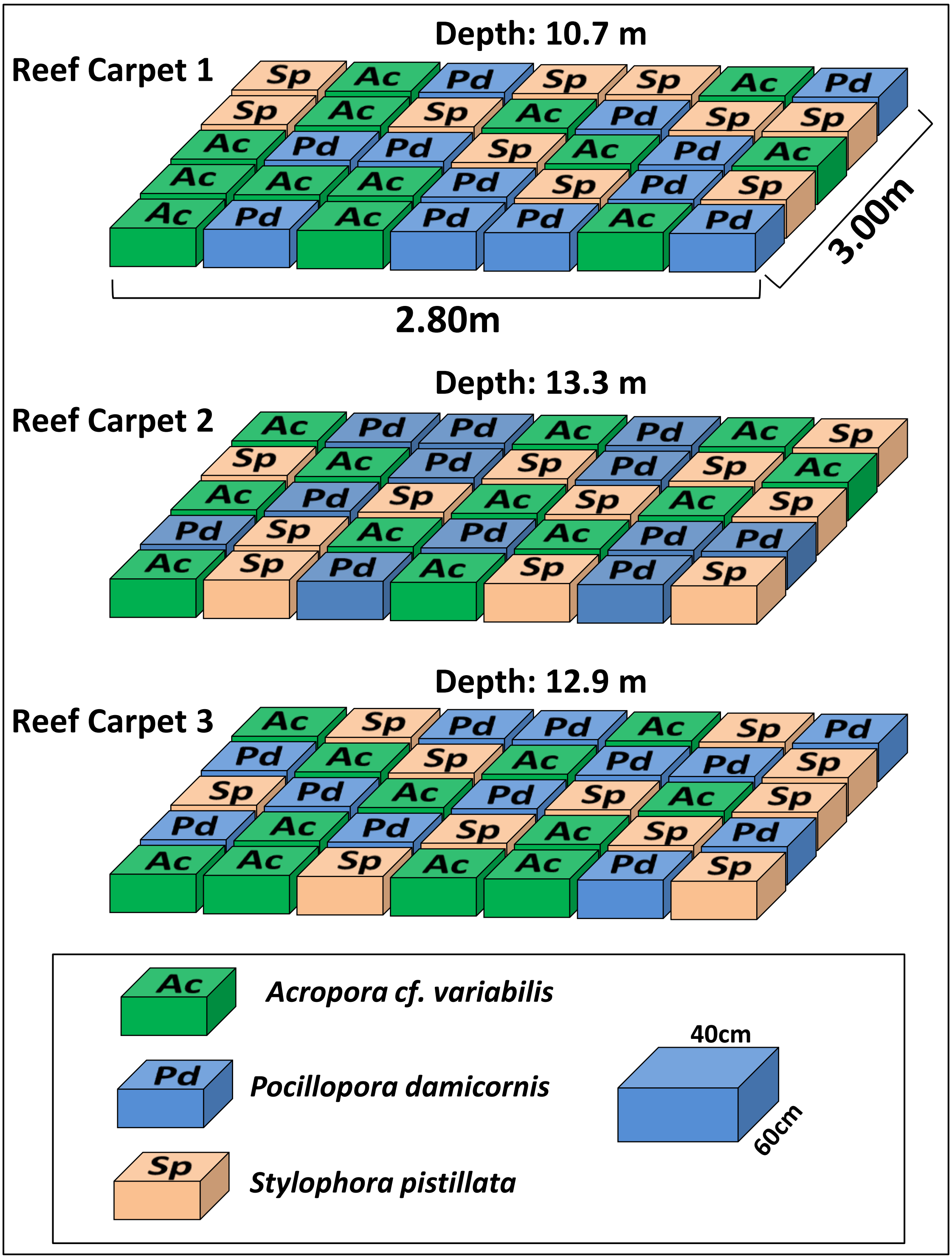

Figure S1:

The experimental design and depth of three Reef Carpets (8.4m^2 each) constructed from 35 preset units (arranged in seven columns and five rows) that were prepared and nursed at the coral nursery. Each unit is made of a nursery-rearing tray (plastic nets of 0.25 cm^2 mesh size, stretched over 40×60cm PVC frames) and includes coral colonies of the same species, along with the biota community that had developed on the tray during the nursery phase.

Fig. S2

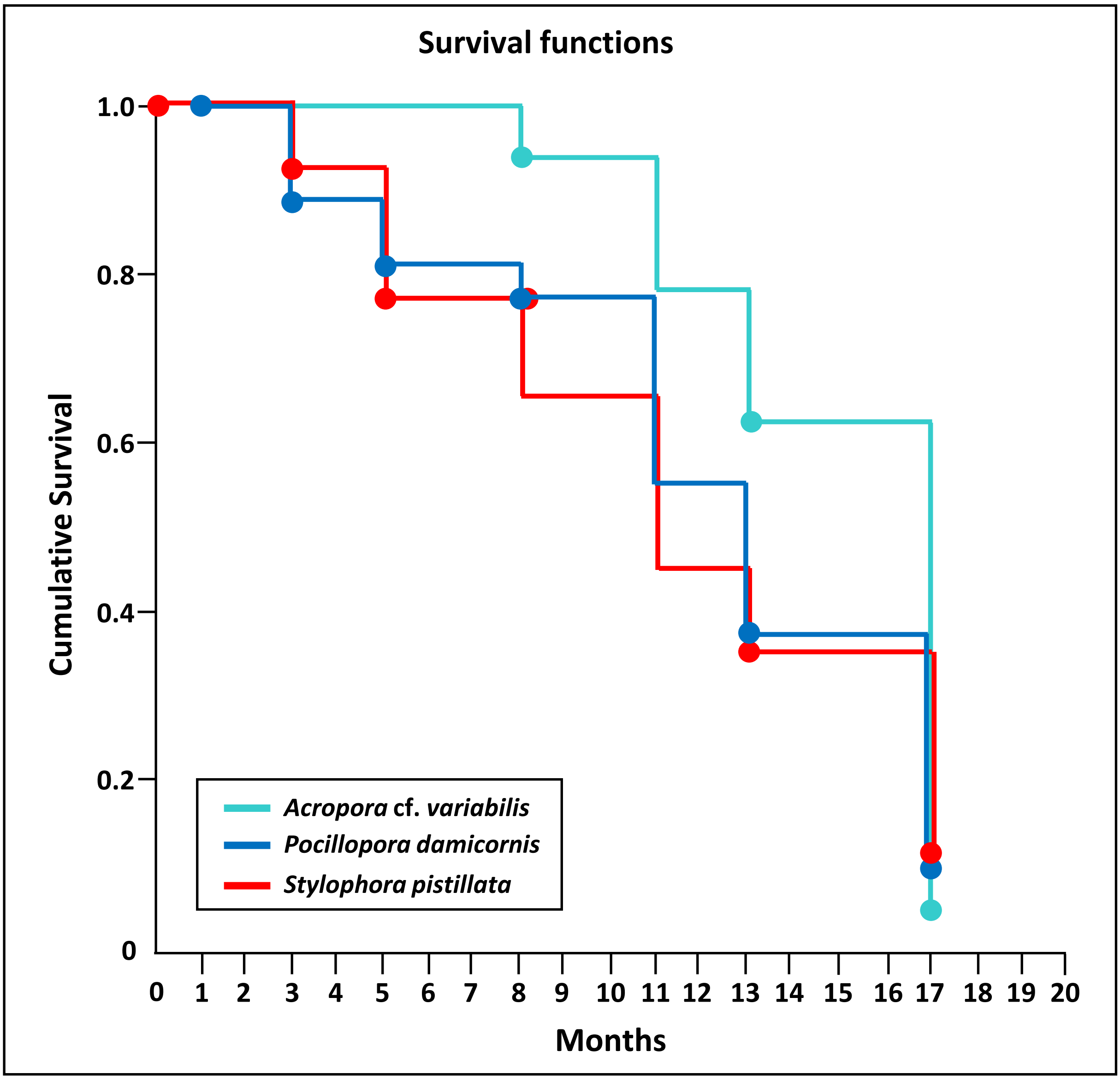

Figure S2:

Kaplan–Meier curve of survival time for *Acropora* cf. *variabilis*, *Pocillopora damicornis* and *Stylophora pistillata* colonies transplanted on a soft-bottom substrate along 17 months. Circles indicate census dates.

Fig. S3

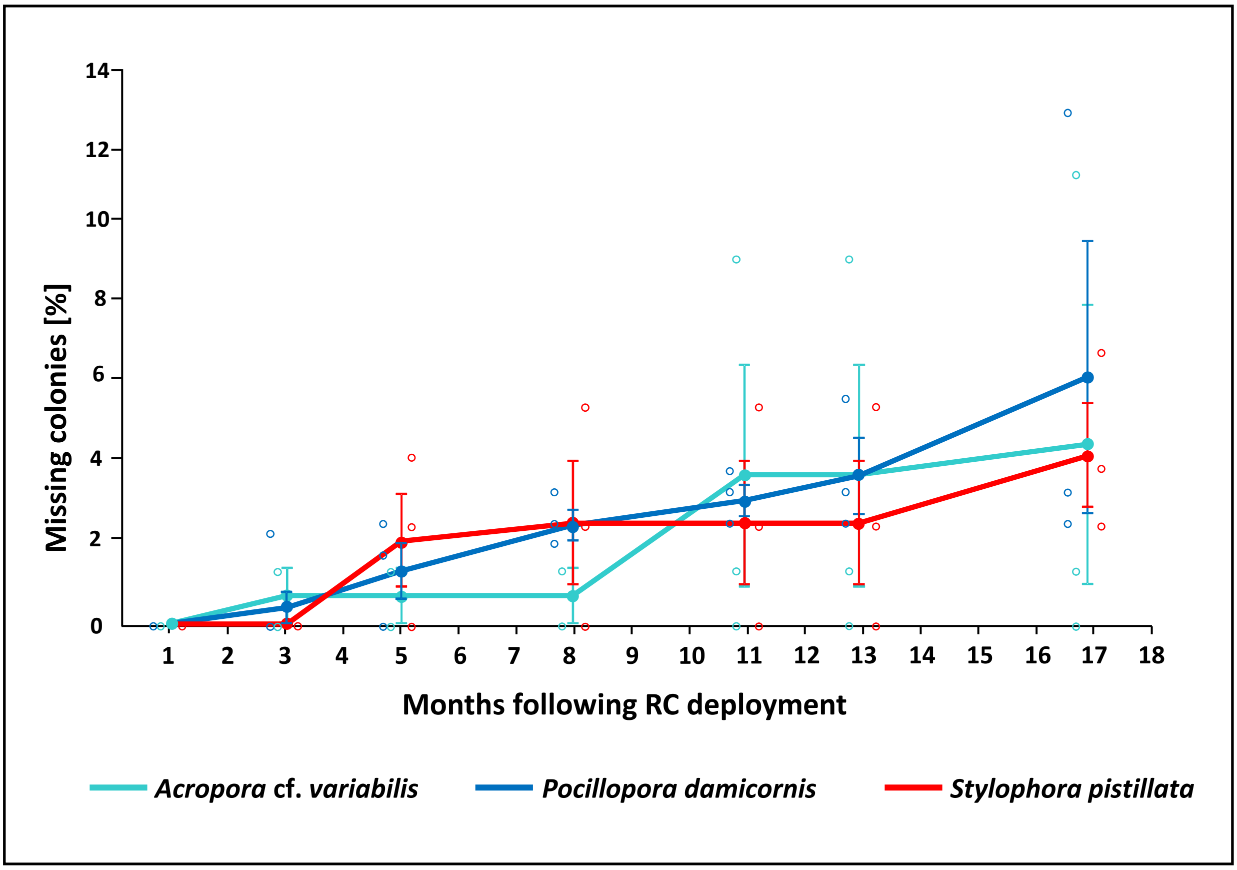

Figure S3:

The percentage of *Acropora* cf. *variabilis*, *Pocillopora damicornis* and *Stylophora pistillata* colonies that disappeared from the Reef Carpets over 17 months (Mean ± SE). No significant effect of time and no significant difference between the three coral species (analysis performed on the proportion of new missing colonies of every observation point; mixed-model ANOVA, $F_{6,36}$=1.131, p=0.364 and $F_{12,36}$=0.746, p=0.698, respectively).

Fig. S4

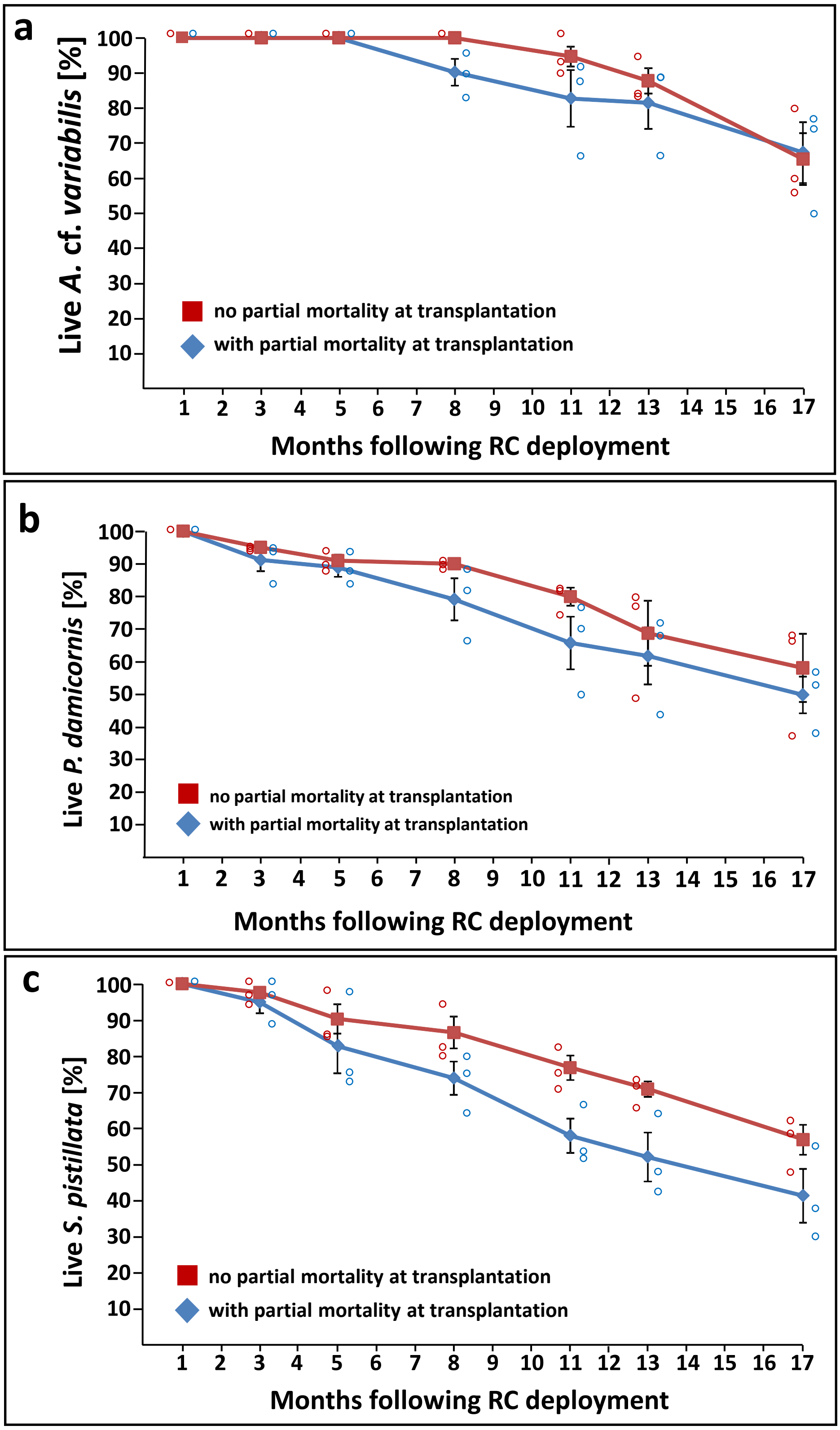

Figure S4:

Comparisons of 17 months survival rates of colonies without, or with partial tissue death at transplantation onset (Mean ± SE). **a** *Acropora* cf. *variabilis*. **b** *Pocillopora damicornis*. **c** *Stylophora pistillata*.

Fig. S5

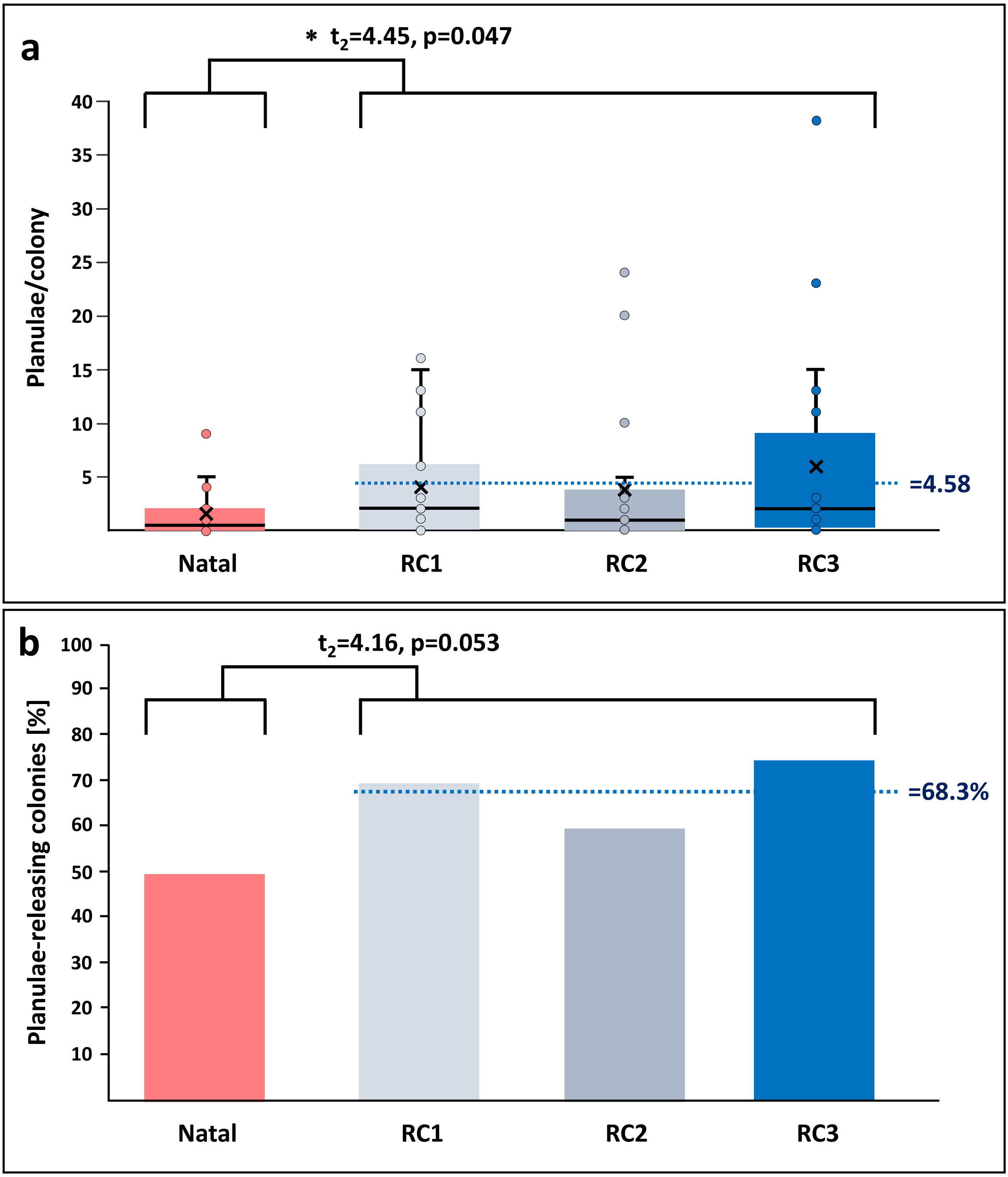

Figure S5:

The reproductive effort of *Stylophora pistillata* colonies at Kisuski Beach. **a** Box plots of the numbers of planulae collected from natal and transplanted colonies. Boxes indicate the lower and upper quartiles, horizontal lines within interquartile range indicate the medians, X indicate the mean, whiskers indicate 1.5x interquartile range. ∗ denotes statistically significant differences (One sample *t*-test; average of three Reef Carpet colonies, RC1-RC3). **b** Percentages of planulae-releasing natal and transplanted colonies (One sample *t*-test; average of three Reef Carpet colonies, RC1-RC3).

Fig. S6

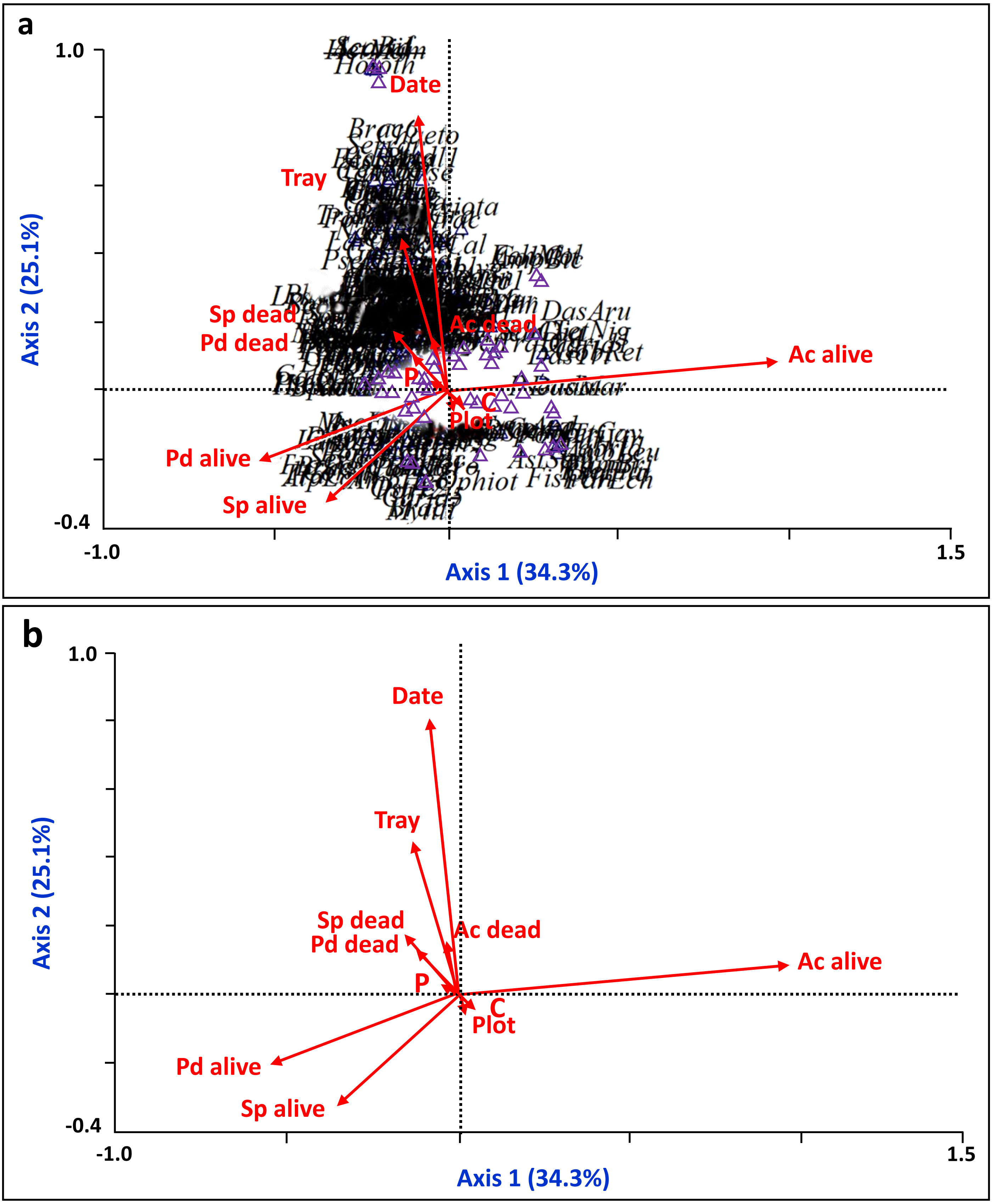

Figure S6:

Species-environment b-plot based on a canonical correspondence analysis (CCA) for the effect of environmental factors (arrows) on RC community structure (species/taxa represented by triangles). **a** The ordination diagram showing both environmental variables and species. **b** For clarity, only the environmental variables are shown. Eigenvalues: axis 1 = 0.507; axis 2 = 0.371. The variance explained by each axis (%) is shown. Environmental variables: time in months since RC transplantation (***Date***); spotted on RC1, RC2 or RC3 (***Plot***); located at the center (***C***) or at the periphery (***P***) of the RC; spotted in a live *Acropora* cf. *variabilis*, *Pocillopora damicornis* or *Stylophora pistillata* colony (***Ac Alive***, ***Pd Alive*** and ***Sp Alive***, respectively), or in a dead colony of the appropriate species (***Ac Dead***, ***Pd Dead*** and ***Sp Dead***, respectively), or at the understory (***Tray***). For list of species abbreviations – see Tables S3 and S4 in the supplementary materials.

Fig. S7

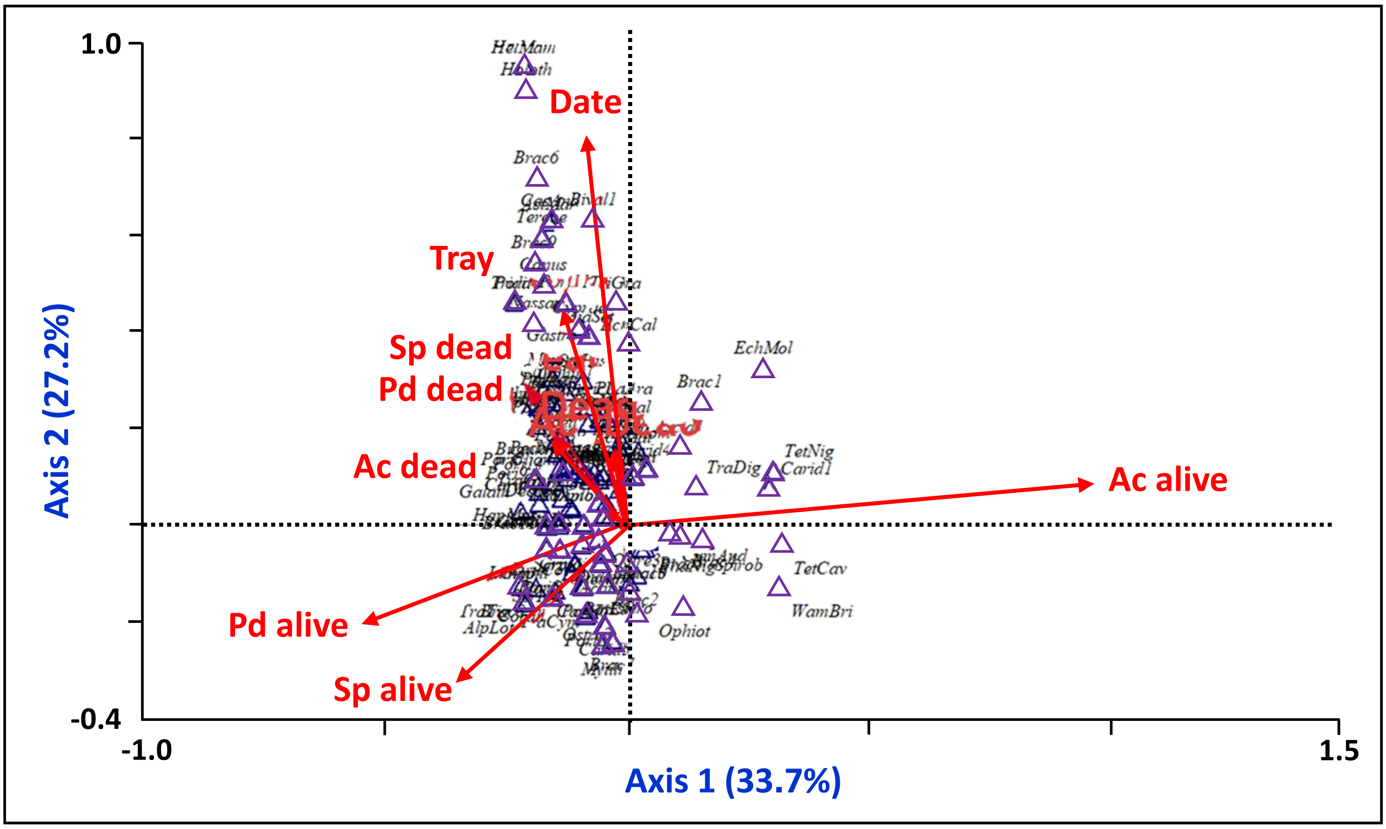

Figure S7:

Species-environment b-plot based on a canonical correspondence analysis (CCA) for the effect of environmental factors (arrows) on the invertebrate community structure (associated species/taxa represented by triangles). Eigenvalues: axis 1 = 0.466; axis 2 = 0.375. The variance explained by each axis (%) is shown. Environmental variables: time in months since RC transplantation (***Date***); spotted in a live *Acropora* cf. *variabilis*, *Pocillopora damicornis* or *Stylophora pistillata* colony (***Ac Alive***, ***Pd Alive*** and ***Sp Alive***, respectively), or in a dead colony of the appropriate species (***Ac Dead***, ***Pd Dead*** and ***Sp Dead***, respectively), or at the understory (***Tray***). For list of species abbreviations – see Table S4 in the supplementary materials.

Fig. S8

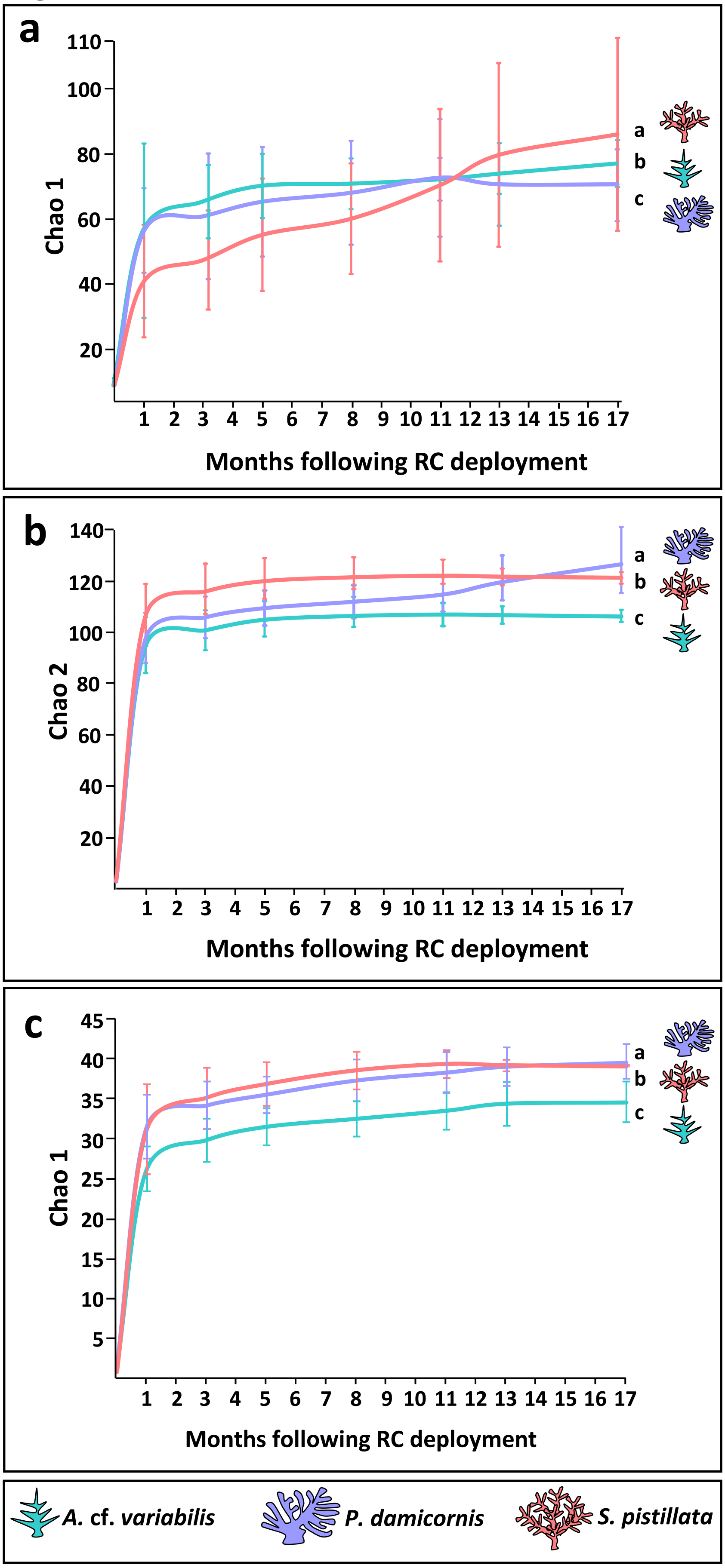

Figure S8 (Species diversity):

The diversity based on Chao 1 and Chao 2 richness estimators (±SD) of the reef-associated communities developing over 17 months at the Reef Carpets. The (a) fish diversity, (b) invertebrate diversity, and (c) crustacean diversity associated with *A.* cf. *variabilis*, *P. damicornis* and *S. pistillata* transplants. In each panel, diversity was compared with one-way ANOVAs. Letters denote statistically significant groups (Tukey HSD Post-hoc tests $p<0.05$; Tables S5, S7, S9).
